# Plasma p-tau181 moderates the association between ten-year atherosclerotic cardiovascular disease risk and mild cognitive impairment – HABS-HD

**DOI:** 10.3389/fnagi.2026.1870150

**Published:** 2026-09-28

**Authors:** Joey A. Contreras, Nancy E. Ortega, Raymond Jones, Roland J. Thorpe, Cellas A. Hayes

**Affiliations:** 1 Department of Neurosciences, Alzheimer’s Disease Cooperative Study (ADCS), University of California, San Diego, San Diego, CA, United States; 2 Department of Medicine, University of Alabama at Birmingham Heersink School of Medicine, Birmingham, AL, United States; 3 Center for Exercise Medicine, University of Alabama at Birmingham, Birmingham, AL, United States; 4 Program for Research on Men’s Health, Johns Hopkins Center for Health Disparities Solutions, Johns Hopkins Bloomberg School of Public Health, Baltimore, MD, United States; 5 Johns Hopkins Alzheimer’s Disease Resource Center for Minority Aging Research, Johns Hopkins Bloomberg School of Public Health, Baltimore, MD, United States; 6 Sanders-Brown Center on Aging, University of Kentucky College of Medicine, Lexington, KY, United States; 7 Department of Neuroscience, University of Kentucky College of Medicine, Lexington, KY, United States

**Keywords:** atherosclerotic cardiovascular disease, biomarker, cardiovascular disease risk, MCI, plasma biomarker, pTau181

## Abstract

**Introduction:**

This study examined cross-sectional associations between ten-year American Heart Association (AHA) atherosclerotic cardiovascular disease (ASCVD) risk and plasma Alzheimer’s disease (AD) biomarkers in relation to cognitive outcomes and to determine whether ASCVD risk modifies biomarker–cognition relationships.

**Methods:**

Baseline data were analyzed from cognitively unimpaired and mild cognitive impaired (MCI) participants enrolled in the Health and Aging Brain Study–Health Disparities (HABS-HD), a large, community-based cohort enriched for racial and ethnic diversity. Plasma biomarkers included amyloid-β42/40 (Aβ42/Aβ40), phosphorylated tau (p-tau181 and p-tau217), and neurofilament light (NfL). Ten-year ASCVD risk was calculated using pooled cohort equations. Multivariable logistic regression models examined associations of ten-year ASCVD risk and plasma biomarkers with MCI, while multivariable linear regression models evaluated associations with Mini-Mental State Examination (MMSE) and Clinical Dementia Rating (CDR) Sum of Boxes scores. Separate models were constructed for Aβ42/40, p-tau217, p-tau181, NfL, and apolipoprotein (*APOE*) ε4 carrier status, and analyses were stratified by race and ethnicity. ASCVD × biomarker interaction terms were tested to evaluate effect modification.

**Results:**

Increased ten-year ASCVD risk was consistently associated with increased odds of MCI, ranging from OR = 2.62 (95% CI: 1.31–5.17) to OR = 4.22 (95% CI: 2.04–8.64), lower MMSE scores, ranging from *β* = −1.90 (95% CI: −2.62 to −1.18) to *β* = −2.56 (95% CI: −3.30 to −1.82), and increased CDR Sum of Boxes scores, ranging from *β* = 0.28 (95% CI: 0.09–0.46) to *β* = 0.39 (95% CI: 0.20–0.58). The ASCVD × p-tau181 interaction was significant for MCI (OR = 1.91; 95% CI: 1.26–2.95) and CDR Sum of Boxes (*β* = 0.27; 95% CI: 0.16–0.39). ASCVD associations with CDR Sum of Boxes ranged from *β* = 0.11 (95% CI: −0.08 to 0.30) to *β* = 0.24 (95% CI: 0.04–0.43) among non-Hispanic White participants, *β* = 0.80 (95% CI: 0.10–1.51) to *β* = 1.17 (95% CI: 0.49–1.86) among non-Hispanic Black participants, and *β* = 0.36 (95% CI: 0.08–0.65) to *β* = 0.52 (95% CI: 0.25–0.80) among Hispanic participants.

**Conclusion:**

Greater ten-year ASCVD risk was consistently associated with cognitive impairment independent of plasma AD biomarkers, with evidence that p-tau181 modifies these associations and that their magnitude varies across racial and ethnic groups.

## Introduction

Alzheimer’s disease (AD) is a progressive neurodegenerative disorder characterized by the accumulation of amyloid-β (Aβ) plaques and hyperphosphorylated tau tangles that ultimately lead to synaptic dysfunction, neuronal loss, and cognitive decline (Hardy and Selkoe, 2002; Braak et al., 2006). Although amyloid and tau pathology define AD biologically, the disease pathology and symptomology is heterogeneous and frequently occurs alongside other age-related processes (Snyder et al., 2015; Jack et al., 2018). Autopsy and neuroimaging studies consistently demonstrate that mixed pathology is common in older adults, particularly within Black persons with AD dementia, with cerebrovascular disease often co-occurring with AD-related changes (Barnes et al., 2015; Kapasi and Schneider, 2016). This overlap may contribute to variability in cognitive presentation, progression, and clinical diagnosis (Almeida et al., 2025; Hayes et al., 2025b; Kapasi et al., 2025; Yu et al., 2026). Despite this convergence, vascular and neurodegenerative mechanisms are often examined separately in studies of cognitive aging.

Atherosclerosis and related cardiometabolic risk factors are well-established contributors to cognitive decline and dementia (Gorelick et al., 2011; Livingston et al., 2024). Hypertension, diabetes, dyslipidemia, and tobacco exposure are highly prevalent in mid- to late-life and have been linked to impaired cerebral perfusion, endothelial dysfunction, and disruption of the blood–brain barrier (Iadecola, 2013a). Epidemiologic studies further demonstrate that composite cardiovascular risk scores predict incident cognitive impairment and dementia (Gottesman et al., 2017; Debette et al., 2019). However, the extent to which systemic atherosclerotic burden interacts with molecular markers of AD pathology remains unclear. This question is particularly relevant given that cardiovascular risk factors are disproportionately represented in populations historically underrepresented in AD research, including Black/African American and Hispanic individuals (Manly et al., 2008; Mayeda et al., 2016), with prior work further highlighting persistent racial disparities in cardiometabolic health and aging outcomes (Thorpe et al., 2016).

Recent advances in blood-based biomarkers allow AD–related biological processes to be assessed using minimally invasive plasma measures (Palmqvist et al., 2020). Plasma biomarkers such as the Aβ42/Aβ40 ratio, phosphorylated tau species (including p-tau 181 and p-tau217), and neurofilament light (NfL) provide indicators of AD pathology, and neuroaxonal injury (Thijssen et al., 2021; Ashton et al., 2022, 2024). Emerging studies have examined associations between cardiovascular risk factors and plasma AD biomarkers (Nation et al., 2019; García-Lluch et al., 2024), with some suggesting additive or synergistic relationships between vascular burden and AD-related processes. However, findings across studies have been inconsistent (Ossenkoppele et al., 2022), and reproducibility across racially and ethnically diverse cohorts remains limited. Differences in disease stage, study design, and operationalization of cardiovascular risk may contribute to variability in reported associations.

Despite rapid progress in blood-based biomarkers for AD, the relationship between cardiovascular risk burden and plasma AD biomarkers during the preclinical and early symptomatic stages of cognitive decline remains incompletely understood. The American Heart Association/American College of Cardiology atherosclerotic cardiovascular disease (ASCVD) risk score integrates multiple cardiometabolic risk factors to estimate ten-year cardiovascular risk and provides a standardized measure of systemic vascular burden and the likelihood of a cardiovascular disease (CVD) event (Arnett et al., 2019). The ASCVD risk score incorporates demographic and cardiometabolic risk factors to estimate an individual’s ten-year risk of major cardiovascular and cerebrovascular events using pooled cohort equations. In this study, ten-year ASCVD risk was used as a composite indicator of cardiovascular and cerebrovascular risk rather than as a direct measure of subclinical atherosclerotic burden. Examining ten-year ASCVD risk alongside plasma AD biomarkers may therefore provide insight into how cumulative vascular risk intersects with AD-related biological processes and influences cognitive impairment in clinically unimpaired and mildly impaired individuals.

Using baseline data from the Health and Aging Brain Study–Health Disparities (HABS-HD), we examined the independent and joint associations of ten-year ASCVD risk, plasma AD biomarkers, and apolipoprotein (*APOE*) ε4 carrier status with cognitive status. Specifically, we aimed to (1) evaluate the cross-sectional associations of ten-year ASCVD risk and individual plasma AD biomarkers with cognitive status among clinically unimpaired and mildly cognitively impaired participants and (2) determine whether ten-year ASCVD risk modifies the associations between plasma AD biomarkers, *APOE* ε4 carrier status, and cognitive status.

## Methods

### HABS-HD

The HABS-HD study previously known as the Health and Aging Brain Among Latino Elders (HABLE) study, is an ongoing longitudinal cohort study based at the Institute for Translational Research at the University of North Texas Health Science Center (O’Bryant et al., 2021; Petersen et al., 2025b). The study was designed as a community-engaged investigation and represents the largest United States cohort focused on AD that intentionally recruits non-Hispanic White (NHW), non-Hispanic Black (NHB), and Hispanic older adults. The HABS-HD study aims to characterize racial and ethnic differences in biological, clinical, and social contributors to AD risk. Participants undergo harmonized evaluations consistent with the amyloid, tau, and neurodegeneration (AT(N)) research framework, including standardized demographic and health questionnaires, comprehensive neuropsychological testing, blood-based biomarker assessments, structural magnetic resonance imaging, amyloid and tau positron emission tomography imaging, and clinician-adjudicated cognitive diagnoses. The study protocol was approved by the University of North Texas Health Science Center Institutional Review Board, and all participants provided written informed consent in accordance with the Declaration of Helsinki.

### Demographic characteristics

Participant age was recorded in years, and educational attainment was quantified as total years of formal schooling completed. Sex was categorized as male or female. Race and ethnicity were self-reported and classified as NHW, NHB, or Hispanic. NHW was used as the reference category.

### APOE genotyping

Genomic DNA was isolated from whole blood samples, and *APOE* genotyping was performed using TaqMan single-nucleotide polymorphism assays targeting rs429358 and rs7412 (Thermo Fisher Scientific) on an Applied Biosystems 7,500 Real-Time PCR platform. Participants were classified as ε4 carriers, defined as those with one or more ε4 alleles, versus non-carriers. *APOE* ε4 carrier status was analyzed as a binary variable (carrier vs. non-carriers) to enhance statistical power and facilitate interpretation of model estimates.

### Blood pressure and blood work

Resting systolic blood pressure was measured in the seated position using two readings obtained during the same study visit and averaged. Total cholesterol and high-density lipoprotein (HDL) cholesterol were quantified from fasting blood samples and expressed in milligrams per deciliter (mg/dL).

### Cardiometabolic risk factors and renal function

Cardiometabolic risk factors included diabetes, hypertension, obesity, and tobacco use, each operationalized as binary variables. Detailed definitions for these measures have been previously described in HABS-HD methodological reports (Hayes et al., 2025a). In brief, hypertension was defined using the HABS-HD CDX_Hypertension variable, which incorporates both prior physician diagnosis and physiologic evidence of elevated blood pressure. Diabetes was defined using the HABS-HD CDX_Diabetes variable, incorporating both physician diagnosis and physiologic evidence of diabetes. Obesity was defined as body mass index (BMI) ≥ 30 kg/m^2^, and tobacco use was defined using the HABS-HD Tobacco Dependence variable (CDX_Tobacco): Consensus Diagnosis—Psychological variable, reflecting current or prior tobacco dependence. Renal function was assessed using a harmonized estimate of glomerular filtration rate (eGFR) calculated using race- and ethnicity-appropriate equations.

### Ten-year ASCVD risk score calculation

Ten-year ASCVD risk was estimated using the American College of Cardiology/American Heart Association Pooled Cohort Equations (PCE) as implemented in the RiskScorescvd R package (version 0.3.1) (Perez-Vicencio et al., 2025). Ten-year ASCVD risk was calculated from baseline study visit data, including age, sex, race and ethnicity, total cholesterol, HDL cholesterol, systolic blood pressure, hypertension status, diabetes status, and tobacco use. Throughout this manuscript, “ten-year ASCVD risk” refers to the predicted probability of an ASCVD event over the subsequent 10 years based on baseline risk factors rather than longitudinal measurements.

Age was entered as a continuous variable. Sex was coded as male or female according to the HABS-HD gender variable. Race and ethnicity were harmonized to match the input categories required by the pooled cohort equation, with NHB participants coded as Black, NHW participants coded as White, and Hispanic participants coded as Other.

Total cholesterol and HDL cholesterol were obtained from HABS-HD blood work variables measured in mg/dL. Because the *RiskScorescvd* package requires cholesterol values in mmol/L, both total cholesterol and HDL cholesterol were converted from mg/dL to mmol/L by multiplying each value by 0.02586.

Systolic blood pressure was calculated using measured blood pressure values collected at the study visit. When two systolic blood pressure measurements were available, the mean of the two values was used; when only one measurement was available, the single available value was used.

Diabetes status was defined using the HABS-HD CDX_Diabetes variable, incorporating both physician diagnosis and physiologic evidence of diabetes (hemoglobin A1c ≥ 6.5%). Tobacco exposure was defined using the HABS-HD CDX_Tobacco variable.

Hypertension status was defined using the HABS-HD CDX_Hypertension variable, which incorporates both prior physician diagnosis and physiologic evidence of hypertension based on measured blood pressure. Participants were classified as hypertensive if they had a documented history of hypertension or persistently elevated blood pressure across study measurements (≥2 readings with systolic blood pressure ≥140 mmHg or diastolic blood pressure ≥90 mmHg). Participants without either criterion were classified as non-hypertensive.

For implementation of the pooled cohort equation, the HABS-HD consensus hypertension variable was used as the hypertension indicator required by the RiskScorescvd package. Measured systolic blood pressure was simultaneously incorporated as the continuous blood pressure component of the equation. Consequently, elevated measured blood pressure contributed directly to ten-year ASCVD risk estimation regardless of self-reported hypertension history. Because a direct antihypertensive treatment-status variable was not available in the format required by the pooled cohort equation, the HABS-HD consensus hypertension variable was used to maximize reproducibility and consistency with the available dataset. We acknowledge that this consensus variable cannot fully distinguish treated, untreated, controlled, and uncontrolled hypertension; therefore, some misclassification of hypertension treatment status is possible.

Both continuous ten-year ASCVD risk scores (used in all statistical analyses) and categorical ten-year ASCVD risk strata (used for descriptive purposes only) were generated.

### Clinical cognitive classification

Cognitive diagnosis in HABS-HD was determined using a diagnostic algorithm followed by consensus review (O’Bryant et al., 2021; Petersen et al., 2025b). The algorithm incorporated self- or informant-reported cognitive change, neuropsychological test performance, and Clinical Dementia Rating Sum of Boxes (CDR-SB) score. Neuropsychological impairment was based on domain-specific *z*-scores, with domain impairment determined from the lowest test scores within each cognitive domain. Domains included attention, executive functioning, language, and memory. Attention was assessed using Digit Span and Trail Making Test Part A; executive functioning using Trail Making Test Part B and Digit Symbol Substitution; language using FAS and animal fluency; and memory using SEVLT delayed recall and WMS-III Logical Memory II. Participants were classified as cognitively unimpaired if they had no self- or informant-reported cognitive change, neuropsychological performance within normal limits, and CDR-SB = 0. Participants were classified as having MCI if they had self- or informant-reported cognitive change, at least one neuropsychological score at or below 1 SD from the normative range, and CDR-SB between 0.5 and 2.0. Participants were classified as having dementia if they had self- or informant-reported cognitive change, at least two neuropsychological scores at or below 2 SD from the normative range, and CDR-SB ≥ 2.5. All cases meeting algorithmic criteria for mild cognitive impairment (MCI) or dementia, as well as 10% of those classified as cognitively unimpaired, underwent consensus review by HABS-HD dementia experts and study staff. In this study, all participants with dementia were excluded.

### Mini-mental state examination

Within the HABS-HD protocol, the Mini-Mental State Examination (MMSE) is administered as part of a comprehensive, standardized neuropsychological battery to assess global cognitive function across diverse racial and ethnic groups. The MMSE evaluates multiple cognitive domains, including orientation, attention, language, visuospatial abilities, and memory. Scores range from 0 to 30, with increasing numerical scores indicating better global cognitive performance.

### Clinical dementia rating sum of boxes

The Clinical Dementia Rating scale was administered using a structured informant interview to assess functional impairment in everyday activities. Within the HABS-HD diagnostic protocol, CDR-SB scores are incorporated into a standardized diagnostic algorithm together with self- or informant-reported cognitive change and neuropsychological test performance to classify participants as cognitively unimpaired, MCI, or dementia. Diagnoses meeting algorithmic criteria are subsequently reviewed through a multidisciplinary consensus process involving dementia experts, study physicians, and clinical staff, consistent with the published HABS-HD methodology and established diagnostic criteria (Petersen et al., 2025b). Participants classified as cognitively unimpaired generally had a CDR-SB score of 0, those with MCI had scores ranging from 0.5 to 2.0, and those with dementia had scores of 2.5 or greater.

### Blood-based AT(N) biomarkers

Blood-based biomarkers representing the AT(N) framework included amyloid-β 40 (Aβ40), amyloid-β 42 (Aβ42), phosphorylated tau 181 (p-tau181), phosphorylated tau 217 (p-tau217), and NfL. Plasma samples were collected under standardized fasting conditions and assayed using single-molecule array (Simoa) technology on the Quanterix HD-X platform, with automated sample handling. All biomarker concentrations were expressed in picograms per milliliter (pg/mL) (Petersen et al., 2025a).

To maximize data completeness and reduce measurement variability across assay waves, biomarker values were harmonized across releases by averaging all available measurements within participants if multiple were available. Specifically, participant-level values for Aβ40, Aβ42, p-tau181, p-tau217, and NfL were calculated as the mean of all non-missing measurements obtained across available assay releases (releases 2, 3, 5, and 7), depending on the biomarker. However, the majority of participants had few duplicate measurements across visits. Aβ42/40 ratios were derived using the averaged Aβ42 and Aβ40 concentrations. Prior to statistical analysis, biomarker distributions were evaluated for extreme values. Extreme outliers were defined *a priori* as observations exceeding five standard deviations above or below the sample mean for each biomarker (Aβ42, Aβ40, p-tau181, p-tau217, and NfL). Values outside this ±5 standard deviation threshold were excluded and coded as missing to minimize undue influence on regression estimates. All plasma biomarkers were transformed prior to modeling to address a right-skewed distribution. Aβ42/40 ratios, p-tau181, p-tau217, and NfL were log2-transformed for regression models to facilitate interpretation per doubling of biomarker concentration for statistical analyses. For analyses requiring concurrent availability of specific biomarkers, the analytic cohort was restricted to participants with non-missing values for p-tau217 and NfL.

### Study specific exclusions

Participants were excluded if they had a history of clinical CVD or met diagnostic criteria for dementia at baseline. Clinical CVD was defined using the HABS-HD CDX_CVD variable, which classifies participants as having CVD if they have a past medical history of myocardial infarction (heart attack), heart failure, cardiomyopathy, atrial fibrillation, or heart valve replacement. Participants with established CVD were excluded because the ten-year ASCVD pooled cohort equation is intended to estimate primary prevention risk among individuals without pre-existing clinical CVD. In addition, analyses were restricted to participants with available measurements for both p-tau217 and NfL.

## Statistical analyses

Participants with complete data for the outcome and primary predictor variables were included in each analysis, with sample sizes varying according to plasma biomarker availability. Plasma biomarkers (Aβ42/40, p-tau217, p-tau181, and NfL) were log2-transformed prior to analysis to normalize distributions and improve model fit. *APOE* ε4 carrier status was analyzed as a binary variable (carrier vs. non-carrier).

### Descriptive statistics

Demographic and clinical characteristics were summarized as mean ± standard deviation for continuous variables and frequency (percentage) for categorical variables.

### Primary regression analyses

Logistic regression models were constructed to examine the independent associations of plasma biomarkers, ten-year ASCVD risk, and *APOE* ε4 carrier status with MCI (cognitively impaired vs. cognitively unimpaired). Five separate models were fit, each including ten-year ASCVD risk and one plasma biomarker as the primary predictors. Odds ratios (ORs) with 95% confidence intervals (CIs) are reported. Model fit was assessed using Tjur’s *R*^2^, Akaike Information Criterion (AIC), and the area under the receiver operating characteristic curve (AUC).

Separate multivariable linear regression models were then constructed to examine associations between plasma biomarkers, ten-year ASCVD risk, and continuous cognitive outcomes, including MMSE total score and CDR-SB score. Five separate models were fit for each cognitive outcome, each including ten-year ASCVD risk and one plasma biomarker as the primary predictors. Regression coefficients (*β*) represent the change in cognitive score per unit increase in each predictor. Model fit was assessed using *R*^2^, adjusted *R*^2^, and AIC.

### Interaction analyses

To determine whether ten-year ASCVD risk modified the associations between plasma biomarkers and cognitive outcomes, multiplicative interaction terms (ASCVD risk × plasma biomarker) were incorporated into both the logistic and linear regression models. Interaction *p*-values <0.05 were considered evidence of effect modification.

### Sensitivity analyses

To evaluate the robustness of the primary findings, regression models were repeated with estimated glomerular filtration rate (eGFR) included as an additional covariate to account for potential confounding by renal function. Models including eGFR were fit among participants with complete renal function data (*N* = 1,990–2,038, depending on biomarker).

### Stratified analyses by race and ethnicity

To examine potential heterogeneity across racial and ethnic groups, all primary linear regression models were repeated separately among NHW, NHB, and Hispanic participants.

### Data visualization

For statistically significant interactions, predicted values from the logistic and linear regression models were generated across the observed range of ten-year ASCVD risk scores, stratified by p-tau181 levels (low = mean −1 SD; high = mean +1 SD). Predicted probabilities (logistic models) and predicted mean cognitive scores (linear models), together with their corresponding 95% CI, were plotted alongside observed data to facilitate interpretation of interaction effects.

Forest plots were generated to display *β* coefficients and 95% CI for all primary predictors across the five plasma biomarker models within each racial and ethnic group. Statistical significance (*p* < 0.05), indicated by confidence intervals that did not cross zero, was denoted with asterisks.

### Statistical significance

A two-tailed significance threshold of *α* = 0.05 was used throughout. Because these analyses were hypothesis-driven, no formal correction for multiple comparisons was applied. Findings should therefore be interpreted within the context of multiple testing.

All analyses were conducted in R (v4.2.3).

## Results

### Descriptive characteristics of the HABS-HD cohort

Table 1 presents the baseline demographic, cardiometabolic, and plasma biomarker characteristics of the analytic sample overall and across ten-year ASCVD risk strata. Among the 2,292 participants included in the analyses (mean age: 64.8 (SD = 8.41), 63.7% female), ten-year ASCVD risk spanned a wide range (median = 0.13; range = 0–0.94). Participants in greater ten-year ASCVD risk categories were progressively older, with mean age increasing from 56.3 ± 4.0 years in the very low-risk group to 72.3 ± 7.2 years in the high-risk group. The proportion of females participants decreased across ten-year ASCVD risk strata (93.7% in the very low-risk group vs. 43.1% in the high-risk group). Cardiometabolic comorbidities increased progressively across ten-year ASCVD risk categories, and plasma biomarkers generally demonstrated increased concentrations with increasing ten-year ASCVD risk. *APOE* ε4 carrier frequency ranged from 21.7 to 29.9% across risk groups without a clear trend. MCI was more prevalent at greater ten-year ASCVD risk, increasing from 16.7% in the very low-risk group to 25.8% in the high-risk group. MMSE total scores and CDR-SB scores are also presented across ten-year ASCVD risk strata.

**Table 1:** Description of the HABS-HD cohort included in the analyses.

	Overall (*N* = 2292)	Very low risk (*N* = 444)	Low risk (*N* = 320)	Moderate risk (*N* = 822)	High risk (*N* = 706)
Ten-year ASCVD risk score, Median [Min, Max]	0.13 [0, 0.94]	0.03 [0, 0.04]	0.06 [0.05, 0.07]	0.13 [0.08, 0.19]	0.30 [0.20, 0.94]
Age (years), Mean (SD)	64.8 (8.4)	56.3 (4.0)	60.3 (5.3)	64.8 (6.2)	72.3 (7.2)
Female, *n* (%)	1459 (63.7)	416 (93.7)	264 (82.5)	475 (57.8)	304 (43.1)
Education (years), Mean (SD)	13.2 (4.4)	13.3 (4.0)	13.2 (4.2)	13.4 (4.2)	13.0 (4.8)
Race and Ethnicity, *n* (%)					
NHW	883 (38.5)	164 (36.9)	100 (31.3)	306 (37.2)	313 (44.3)
NHB	473 (20.6)	52 (11.7)	78 (24.4)	213 (25.9)	130 (18.4)
Hispanic	936 (40.8)	228 (51.4)	142 (44.4)	303 (36.9)	263 (37.3)
Diabetes, *n* (%)	539 (23.5)	29 (6.5)	52 (16.3)	172 (20.9)	286 (40.5)
Hypertension, *n* (%)	1444 (63.0)	116 (26.1)	155 (48.4)	573 (69.7)	600 (85.0)
Tobacco Dependence, *n* (%)	154 (6.7)	6 (1.4)	16 (5.0)	56 (6.8)	76 (10.8)
SBP (mmHg), Mean (SD)	135 (18.8)	121 (13.5)	130 (14.3)	136 (16.6)	146 (19.1)
Total cholesterol (mmol/L), Mean (SD)	4.7 (1.1)	5.0 (1.0)	4.9 (1.0)	4.7 (1.0)	4.5 (1.1)
Total HDL (mmol/L), Mean (SD)	1.4 (0.4)	1.6 (0.4)	1.5 (0.4)	1.4 (0.4)	1.3 (0.4)
APOE ε4, *n* (%)	613 (26.7)	128 (28.8)	86 (26.9)	246 (29.9)	153 (21.7)
Missing, *n* (%)	32 (1.4)	8 (1.8)	4 (1.3)	12 (1.5)	8 (1.1)
eGFR, Mean (SD)	82.2 (16.9)	90.6 (13.5)	88.9 (14.3)	81.6 (15.9)	75.0 (17.5)
Missing, *n* (%)	254 (11.1)	61 (13.7)	41 (12.8)	80 (9.7)	72 (10.2)
Aβ4240 (pg/mL), Median [Min, Max]	0.05 [0.01, 0.3]	0.05 [0.01, 0.3]	0.05 [0.02, 0.1]	0.05 [0.02, 0.1]	0.05 [0.01, 0.2]
Missing, *n* (%)	52 (2.3)	5 (1.1)	6 (1.9)	23 (2.8)	18 (2.5)
p-tau217 (pg/mL), Median [Min, Max]	0.3 [0.1, 1.8]	0.2 [0.1, 1.7]	0.3 [0.1, 1.4]	0.3 [0.1, 1.7]	0.4 [0.1, 1.8]
p-tau181 (pg/mL), Median [Min, Max]	2.1 [0.5, 85.8]	1.7 [0.5, 38.1]	1.9 [0.6, 42.7]	2.0 [0.5, 63.2]	2.5 [0.6, 85.8]
Missing, *n* (%)	69 (3.0)	7 (1.6)	7 (2.2)	33 (4.0)	22 (3.1)
NfL (pg/mL), Median [Min, Max]	12.6 [1.7, 80.6]	9.4 [2.7, 56.3]	10.9 [2.4, 58.2]	12.3 [2.4, 78.9]	17.4 [1.7, 80.6]
Cognitive Status, *n* (%)					
Unimpaired	1805 (78.8)	370 (83.3)	247 (77.2)	664 (80.8)	524 (74.2)
Mild Cognitive Impairment	487 (21.2)	74 (16.7)	73 (22.8)	158 (19.2)	182 (25.8)
MMSE Total					
Mean (SD)	27.8 (2.5)	28.0 (2.5)	27.9 (2.5)	28.0 (2.2)	27.4 (2.8)
Median [Min, Max]	29.0 [12.0, 30.0]	29.0 [12.0, 30.0]	29.0 [12.0, 30.0]	29.0 [14.0, 30.0]	28.0 [14.0, 30.0]
Missing, *n* (%)	10 (0.4)	3 (0.7)	1 (0.3)	5 (0.6)	1 (0.1)
CDR Sum of Boxes					
Mean (SD)	0.26 (0.6)	0.19 (0.49)	0.27 (0.65)	0.24 (0.62)	0.33 (0.7)
Median [Min, Max]	0 [0, 5.0]	0 [0, 3.5]	0 [0, 4.5]	0 [0, 5.0]	0 [0, 4.0]

Values are presented as mean (standard deviation, SD), median [minimum, maximum], or n (%), as appropriate. Participants are categorized into ASCVD risk groups (very low, low, moderate, and high) based on 10-year risk estimates, with overall totals shown. Biomarker values (Aβ42/Aβ40, p-tau217, p-tau181, and neurofilament light [NfL]) are reported as medians due to skewed distributions. Missing data are reported for applicable variables. NHW, non-Hispanic White; NHB, non-Hispanic Black; HDL, high-density lipoprotein; eGFR, estimated glomerular filtration rate; MMSE, mini mental state examination; CDR, clinical dementia rating

### Plasma biomarker and ten-year ASCVD risk associations with mild cognitive impairment

Logistic regression models examining MCI risk revealed significant associations with both ten-year ASCVD risk and specific plasma biomarkers (Table 2). Ten-year ASCVD risk was consistently associated with increased odds of MCI across all five models, with ORs ranging from 2.62 (95% CI: 1.31 to 5.17; *p* = 0.006) to 4.22 (95% CI: 2.04 to 8.64; *p* < 0.001). Among the plasma biomarkers examined, p-tau181 was associated with increased odds of MCI (OR = 1.22; 95% CI: 1.15 to 1.30; *p* < 0.001), indicating that each log2-unit increase in p-tau181 was associated with 22% increased odds of MCI. Increases in plasma p-tau217 was also associated with increased odds of MCI (OR = 1.15; 95% CI: 1.00 to 1.31; *p* = 0.043), whereas increases in Aβ42/40 was associated with lower odds of MCI (OR = 0.69; 95% CI: 0.53 to 0.89; *p* = 0.004). NfL and *APOE* ε4 carrier status were not significantly associated with MCI in the primary.

**Table 2:** Logistic regression models examining associations between plasma biomarkers, ten-year atherosclerotic cardiovascular disease risk, and mild cognitive impairment.

Variable	*p*-value	OR (95% CI)	*R*^2^ Tjur	AIC	AUC	VIF
**Model 1: Aβ42/40 + ten-year ASCVD risk score (*N* = 2240)**			0.009	2301.429	0.561	
Aβ42/40 (log2)	0.004	0.69 (0.53 to 0.89)				1.002
Ten-year ASCVD risk score	0.001	2.98 (1.53 to 5.74)				1.002
**Model 2: p-Tau217 + ten-year ASCVD risk score (*N* = 2292)**			0.007	2360.602	0.556	
p-Tau217 (log2)	0.043	1.15 (1.00 to 1.31)				1.104
Ten-year ASCVD risk score	0.006	2.62 (1.31 to 5.17)				1.104
**Model 3: p-Tau181 + ten-year ASCVD risk score (*N* = 2223)**			0.026	2255.368	0.602	
p-Tau181 (log2)	<0.001	1.22 (1.15 to 1.30)				1.000
Ten-year ASCVD risk score	0.002	2.86 (1.46 to 5.53)				1.000
**Model 4: NfL + ten-year ASCVD risk score (*N* = 2292)**			0.007	2362.048	0.558	
NfL (log2)	0.105	0.89 (0.77 to 1.02)				1.234
Ten-year ASCVD risk score	<0.001	4.22 (2.04 to 8.64)				1.234
**Model 5: APOE ε4 + ten-year ASCVD risk score (*N* = 2260)**			0.007	2333.412	0.558	
APOE ε4	0.140	1.18 (0.94 to 1.48)				1.008
Ten-year ASCVD risk score	<0.001	3.54 (1.83 to 6.80)				1.008

Logistic regression models examining the independent associations of five plasma biomarkers and ten-year atherosclerotic cardiovascular disease (ASCVD) risk with mild cognitive impairment (MCI) status. Each model includes one plasma biomarker alongside ten-year ASCVD risk score as a covariate. Model 1 includes Aβ42/40 (log2-transformed); Model 2 includes p-tau217 (log2-transformed); Model 3 includes p-tau181 (log2-transformed); Model 4 includes neurofilament light chain (NfL, log2-transformed); and Model 5 includes apolipoprotein E ε4 carrier status (APOE ε4, binary). Odds ratios (OR) represent the change in odds of MCI per unit increase in the biomarker (or presence of APOE ε4 carrier status). Sample sizes (*N*) are provided for each model and vary due to missing biomarker data. *R*^2^ Tjur represents the coefficient of determination for logistic regression. AIC (Akaike Information Criterion) and AUC (area under the receiver operating characteristic curve) assess model fit. VIF (variance inflation factor) tests for multicollinearity. *p*-values <0.05 indicate statistical significance.

### Sensitivity analysis: adjustment for estimated glomerular filtration rate

When eGFR was included as an additional covariate, ten-year ASCVD risk associations with MCI remained substantial and statistically significant (OR ranging from OR = 3.36; 95% CI: 1.54 to 7.27 to OR = 4.87, 95% CI: 2.18 to 10.79) (Supplementary Table 1). The inclusion of eGFR did not substantially attenuate ten-year ASCVD or biomarker associations.

### Plasma biomarker and ten-year ASCVD risk associations with global cognition (MMSE)

Beyond MCI status, increasing ten-year ASCVD risk was consistently associated with lower MMSE total scores (Table 3). In models adjusted for individual plasma biomarkers, increases in ten-year ASCVD scores was associated with lower MMSE scores; Model 1 Aβ42/40: *β* = −1.90, 95% CI: −2.62 to −1.18) to *β* = −2.56 (95% CI: −3.30 to −1.82; Model 2, p-tau217). Among the plasma biomarkers examined, increases in p-tau217 was associated with increasing MMSE scores (*β* = 0.32; 95% CI: 0.18 to 0.45; *p* < 0.001), and increases in p-tau181 was associated with lower MMSE scores (*β* = −0.06; 95% CI: −0.13 to −0.00; *p* = 0.045). Aβ42/40 and NfL did not reach statistical significance. *APOE ε4* carrier status was also associated with increasing MMSE scores (*β* = 0.45; 95% CI: 0.22 to 0.69; *p* < 0.001).

**Table 3:** Linear regression models examining associations between plasma biomarkers, 10 year atherosclerotic cardiovascular disease risk, and Mini-Mental State Examination (MMSE) scores.

Variable	*p*-value	*β* (95% CI)	*R*^2^/Adjusted *R*^2^	AIC	VIF
**Model 1: Aβ42/40 + ten-year ASCVD risk score (*N* = 2231)**			0.014 / 0.013	10434.105	
Aβ42/40 (log2)	0.107	0.22 (−0.05 to 0.49)			1.003
Ten-year ASCVD risk score	<0.001	−1.90 (−2.62 to −1.18)			1.003
**Model 2: p-Tau217 + ten-year ASCVD risk score (*N* = 2282)**			0.023 / 0.022	10644.870	
p-Tau217 (log2)	<0.001	0.32 (0.18 to 0.45)			1.100
Ten-year ASCVD risk score	<0.001	−2.56 (−3.30 to −1.82)			1.100
**Model 3: p-Tau181 + ten-year ASCVD risk score (*N* = 2213)**			0.015 / 0.014	10286.378	
p-Tau181 (log2)	0.045	−0.06 (−0.13 to −0.00)			1.003
Ten-year ASCVD risk score	<0.001	−1.90 (−2.61 to −1.20)			1.003
**Model 4: NfL + ten-year ASCVD risk score (*N* = 2282)**			0.014 / 0.013	10665.209	
NfL (log2)	0.606	0.04 (−0.11 to 0.18)			1.209
Ten-year ASCVD risk score	<0.001	−2.13 (−2.91 to −1.35)			1.209
**Model 5: APOE ε4 + ten-year ASCVD risk score (*N* = 2250)**			0.021 / 0.020	10498.421	
APOE ε4	<0.001	0.45 (0.22 to 0.69)			1.006
Ten-year ASCVD risk score	<0.001	−1.99 (−2.71 to −1.28)			1.006

Linear regression models examining the independent associations of five plasma biomarkers and ten-year atherosclerotic cardiovascular disease (ASCVD) risk with global cognitive function assessed by Mini-Mental State Examination (MMSE) total score. Each model includes one plasma biomarker alongside ten-year ASCVD risk score as a covariate. Model 1 includes Aβ42/40 (log2-transformed); Model 2 includes p-tau217 (log2-transformed); Model 3 includes p-tau181 (log2-transformed); Model 4 includes neurofilament light chain (NfL, log2-transformed); and Model 5 includes apolipoprotein E ε4 carrier status (APOE ε4, binary). *β* coefficients represent the change in MMSE score per unit increase in the biomarker (or presence of APOE ε4 carrier status). Sample sizes (*N*) are provided for each model and vary due to missing biomarker data. *R*^2^, coefficient of determination; Adjusted *R*^2^, *R*^2^ adjusted for number of predictors; AIC, Akaike Information Criterion; VIF, variance inflation factor (tests for multicollinearity). *p*-values <0.05 indicate statistical significance.

### Plasma biomarker and ten-year ASCVD risk associations with clinical dementia rating sum of boxes scores

Associations between plasma biomarkers and dementia severity, assessed by CDR-SB score, revealed differential effects across the five biomarker models (Table 4). Ten-year ASCVD risk was consistently associated with increasing CDR-SB scores, indicating greater dementia severity. Ten-year ASCVD β coefficients ranged from 0.28 (95% CI: 0.09 to 0.46; *p* = 0.003) in the p-tau217 model to 0.39 (95% CI: 0.20 to 0.58; *p* < 0.001) in the NfL model. Among the five biomarkers examined, p-tau181 was significantly associated with dementia severity (*β* = 0.06; 95% CI: 0.04 to 0.08; *p* < 0.001). Plasma p-tau217 also showed a significant positive association (*β* = 0.05, 95% CI: 0.02 to 0.08; *p* = 0.003). Aβ42/40 demonstrated an inverse association with dementia severity (*β* = −0.10; 95% CI: −0.16 to −0.03; *p* = 0.003), indicating increasing Aβ42/40 levels associated with less severe dementia. NfL and *APOE* ε4 did not reach statistical significance.

**Table 4:** Linear regression models examining associations between plasma biomarkers, 10 year atherosclerotic cardiovascular disease risk, and Clinical Dementia Rating Sum of Boxes scores.

Variable	*p*-value	*β* (95% CI)	*R*^2^/Adjusted *R*^2^	AIC	VIF
**Model 1: Aβ42/40 + ten-year ASCVD risk score (*N* = 2240)**			0.010 / 0.009	4161.513	
Aβ42/40 (log2)	0.003	−0.10 (−0.16 to −0.03)			1.003
Ten-year ASCVD risk score	<0.001	0.33 (0.15 to 0.50)			1.003
**Model 2: p-Tau217 + ten-year ASCVD risk score (*N* = 2292)**			0.011 / 0.010	4317.990	
p-Tau217 (log2)	0.003	0.05 (0.02 to 0.08)			1.102
Ten-year ASCVD risk score	0.003	0.28 (0.09 to 0.46)			1.102
**Model 3: p-Tau181 + ten-year ASCVD risk score (*N* = 2223)**			0.032 / 0.031	4122.315	
p-Tau181 (log2)	<0.001	0.06 (0.04 to 0.08)			1.003
Ten-year ASCVD risk score	0.001	0.30 (0.12 to 0.47)			1.003
**Model 4: NfL + ten-year ASCVD risk score (*N* = 2292)**			0.007 / 0.006	4326.050	
NfL (log2)	0.483	−0.01 (−0.05 to 0.02)			1.209
Ten-year ASCVD risk score	<0.001	0.39 (0.20 to 0.58)			1.209
**Model 5: APOE ε4 + ten-year ASCVD risk score (*N* = 2260)**			0.008 / 0.008	4275.627	
APOE ε4	0.146	0.04 (−0.01 to 0.10)			1.006
Ten-year ASCVD risk score	<0.001	0.38 (0.21 to 0.56)			1.006

Linear regression models examining the independent associations of five plasma biomarkers and ten-year atherosclerotic cardiovascular disease (ASCVD) risk with global cognitive function assessed by Clinical Dementia Rating (CDR) Sum of Boxes score. Each model includes one plasma biomarker alongside ten-year ASCVD risk score as a covariate. Model 1 includes Aβ42/40 (log2-transformed); Model 2 includes p-tau217 (log2-transformed); Model 3 includes p-tau181 (log2-transformed); Model 4 includes neurofilament light chain (NfL, log2-transformed); and Model 5 includes apolipoprotein E ε4 carrier status (APOE ε4, binary). *β* coefficients represent the change in MMSE score per unit increase in the biomarker (or presence of APOE ε4 carrier status). Sample sizes (*N*) are provided for each model and vary due to missing biomarker data. *R*^2^, coefficient of determination; Adjusted *R*^2^, *R*^2^ adjusted for number of predictors; AIC, Akaike Information Criterion; VIF, variance inflation factor (tests for multicollinearity). *p*-values <0.05 indicate statistical significance.

### Interaction between p-tau181 and ten-year ASCVD risk on cognitive outcomes

Examination of interactions revealed significant effect modification of ten-year ASCVD risk associations by p-tau181 levels on two of the three cognitive outcome measures. For MCI risk, the ten-year ASCVD × p-tau181 interaction term reached statistical significance (OR = 1.91; 95% CI: 1.26 to 2.95; *p* = 0.003) (Table 5, Figure 1A). For MMSE scores, the ten-year ASCVD × p-tau181 interaction was not significant (*β* = 0.12; 95% CI: −0.34 to 0.57; *p* = 0.62) (Figure 1B, Supplementary Table 2). For CDR-SB, the interaction reached statistical significance (*β* = 0.27; 95% CI: 0.16 to 0.39; *p* < 0.001), indicating that p-tau181 modified the association between ten-year ASCVD risk and dementia severity (Figure 1C, Supplementary Table 3). No significant interactions were detected for Aβ42/40, p-tau217, NfL, or *APOE* ε4 across the three cognitive outcomes (Table 5: MCI as the outcome; Supplementary Table 2: MMSE Scores as the outcome; Supplementary Table 3: CDR-SB as the outcome).

**Table 5:** Logistic regression interaction models examining ASCVD risk × plasma biomarker interactions in relation to mild cognitive impairment.

Variable	*p*-value	OR (95% CI)	*R* ^2^	AIC	AUC	VIF
**Model 1: Aβ42/40 + ten-year ASCVD risk score + Interaction (*N* = 2240)**			0.010	2302.492	0.558	
Aβ42/40 (log2)	0.266	0.80 (0.53 to 1.19)				2.399
Ten-year ASCVD risk score	0.499	0.07 (0.00 to 135.82)				130.649
Ten-year ASCVD risk score × Aβ42/40 (log2)	0.335	0.44 (0.08 to 2.33)				133.646
**Model 2: p-Tau217 + ten-year ASCVD risk score + Interaction (*N* = 2292)**			0.007	2362.597	0.556	
p-Tau217 (log2)	0.195	1.14 (0.94 to 1.39)				2.506
Ten-year ASCVD Risk	0.110	2.71 (0.78 to 9.19)				3.540
Ten-year ASCVD risk score × p-Tau217 (log2)	0.946	1.03 (0.48 to 2.18)				3.697
**Model 3: p-Tau181 + ten-year ASCVD risk score + Interaction (*N* = 2223)**			0.032	2248.182	0.606	
p-Tau181 (log2)	0.049	1.10 (1.00 to 1.20)				2.414
Ten-year ASCVD risk score	0.632	0.76 (0.25 to 2.27)				2.619
Ten-year ASCVD risk score × p-Tau181 (log2)	0.003	1.91 (1.26 to 2.95)				4.230
**Model 4: NfL + ten-year ASCVD risk score + Interaction (*N* = 2292)**			0.007	2364.001	0.558	
NfL (log2)	0.322	0.90 (0.74 to 1.10)				2.467
Ten-year ASCVD risk score	0.293	6.07 (0.21 to 177.66)				26.747
Ten-year ASCVD risk score × NfL (log2)	0.828	0.92 (0.41 to 2.03)				32.608
**Model 5: APOE ε4 + ten-year ASCVD risk score + Interaction (*N* = 2260)**			0.007	2335.226	0.559	
APOE ε4	0.526	1.12 (0.79 to 1.57)				2.377
Ten-year ASCVD risk score	0.002	3.28 (1.54 to 6.87)				1.298
Ten-year ASCVD risk score × APOE ε4	0.666	1.41 (0.29 to 6.81)				2.562

Logistic regression models with interaction terms examining whether the associations between plasma biomarkers and mild cognitive impairment (MCI) are modified by ten-year atherosclerotic cardiovascular disease (ASCVD) risk. Each model includes one plasma biomarker, ten-year ASCVD risk score, and the interaction term (ASCVD × biomarker). Model 1 includes Aβ42/40 (log2-transformed); Model 2 includes p-tau217 (log2-transformed); Model 3 includes p-tau181 (log2-transformed); Model 4 includes neurofilament light chain (NfL, log2-transformed); and Model 5 includes apolipoprotein E ε4 carrier status (APOE ε4, binary). Odds ratios (OR) represent the change in odds of MCI per unit increase in the respective predictor. Sample sizes (*N*) are provided for each model. *R*^2^ Tjur represents the coefficient of determination for logistic regression. AIC (Akaike Information Criterion) and AUC (area under the receiver operating characteristic curve) assess model fit. VIF (variance inflation factor) tests for multicollinearity; elevated VIF values (>5) in Models 1 and 4 indicate substantial collinearity likely introduced by the interaction terms. *p*-values <0.05 indicate statistical significance. OR, odds ratio; CI, confidence interval; R2 = Tjur’s R2; AIC, Akaike Information Criterion; AUC, area under the curve; VIF, variance inflation factor. OR, odds ratio; CI, confidence interval; *R*^2^ = Tjur’s *R*^2^; AIC, Akaike Information Criterion; AUC, area under the curve; VIF, variance inflation factor.

**Figure 1:**
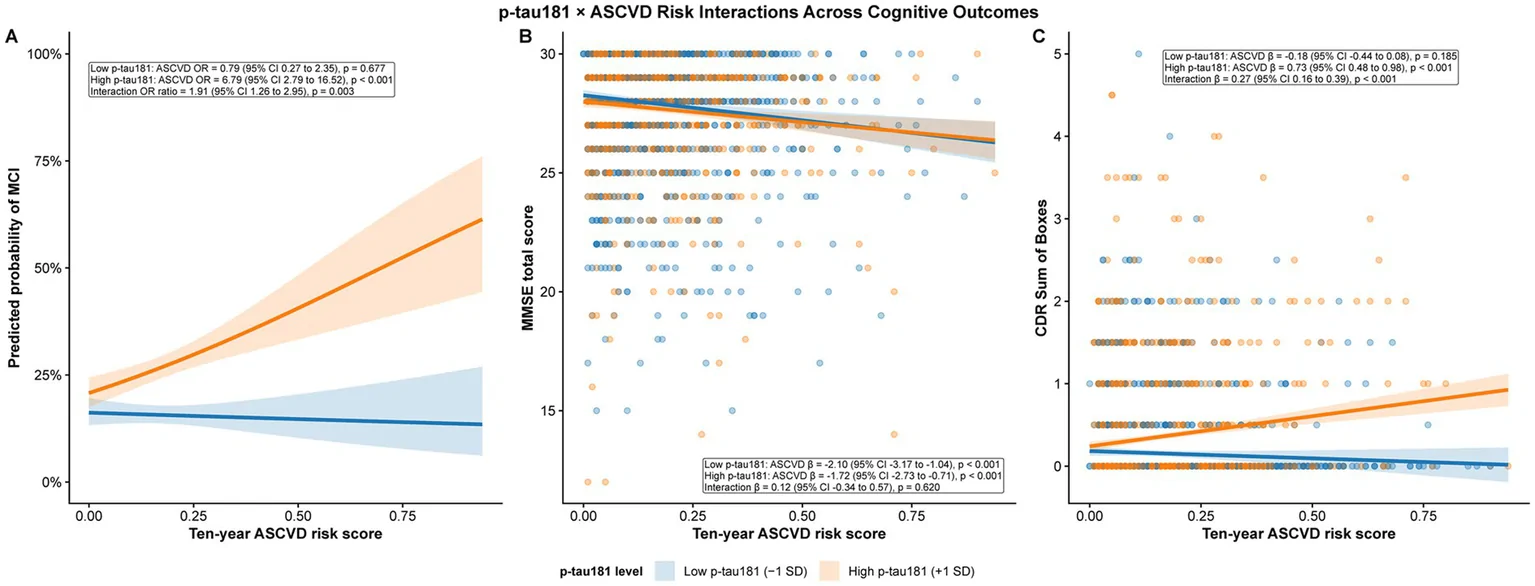
Interaction effects of p-tau181 and 10-year atherosclerotic cardiovascular disease risk on cognitive impairment and cognitive performance. Graphical representation of the interaction between p-tau181 and 10-year atherosclerotic cardiovascular disease (ASCVD) risk on three cognitive outcomes. p-tau181 was dichotomized at ±1 standard deviation (SD) from the mean to illustrate differential associations at low (−1 SD; blue) and high (+1 SD; orange) levels. Predicted values were generated from fitted interaction models. Shaded regions represent 95% confidence intervals around point estimates. Coefficient estimates (*β*) and interaction *p*-values are displayed with the plots. **(A)** Logistic regression model showing the predicted probability of Mild Cognitive Impairment (MCI) across 10-year ASCVD risk scores, stratified by p-tau181 level. **(B)** Linear regression model showing Mini-Mental State Examination (MMSE) total scores across 10-year ASCVD risk scores, stratified by p-tau181 level. Individual data points (circles) are jittered for visibility. **(C)** Linear regression model showing Clinical Dementia Rating (CDR) Sum of Boxes scores across 10 year ASCVD risk scores, stratified by p-tau181 level. Individual data points (circles) represent observed dementia severity. All models include ASCVD score, p-tau181 (log2-transformed), and their multiplicative interaction term.

### Race and ethnic stratifications for MMSE associations

Among NHW participants (*N* = 854–877), ten-year ASCVD risk was consistently associated with lower MMSE scores across all five models, with *β* coefficients ranging from −1.62 (95% CI: −2.08 to −1.17; Model 1) to −1.96 (95% CI: −2.48 to −1.43; Model 4) (Figure 2, Supplementary Table 4). Aβ42/40 and *APOE* ε4 carrier status was not associated with MMSE in this stratum. Among NHB participants (*N* = 451–472), ten-year ASCVD regression coefficients were greater in magnitude than those observed in NHW participants. ASCVD risk *β* coefficients ranged from −2.14 (95% CI: −3.68 to −0.60; *p* = 0.006) to −2.47 (95% CI: −4.03 to −0.91; *p* = 0.002) (Figure 2, Supplementary Table 5). Notably, both p-tau181 (*β* = −0.14; 95% CI: −0.22 to −0.05; *p* < 0.001) and NfL (*β* = −0.33; 95% CI: −0.54 to −0.13; *p* = 0.002) demonstrated significant inverse associations with MMSE in NHB participants. Hispanic participants (*N* = 890–933) demonstrated the largest ten-year ASCVD regression coefficients for MMSE, with *β* coefficients ranging from −3.26 (95% CI: −4.72 to −1.80; Model 4) to −3.90 (95% CI: −5.29 to −2.52; Model 2), all reaching *p* < 0.001 (Figure 2, Supplementary Table 6). Across all three race and ethnicity strata, ten-year ASCVD risk demonstrated an inverse association with MMSE scores, with Hispanic participants showing approximately two-fold greater magnitude of ten-year ASCVD effect relative to NHW participants (Figure 2, Supplementary Tables 4–6).

**Figure 2:**
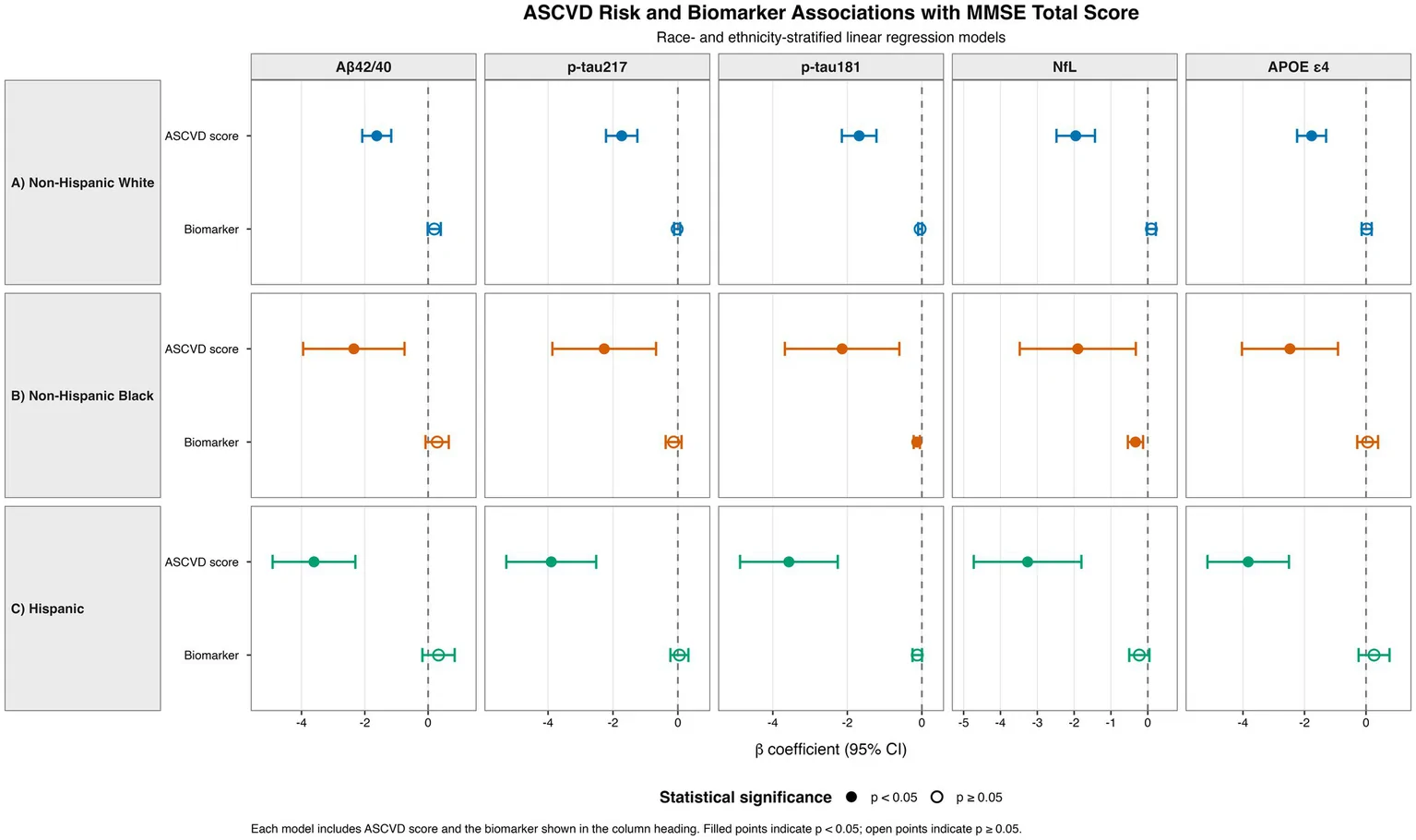
Forest plots of plasma biomarker and 10-year atherosclerotic cardiovascular disease (ASCVD) risk associations with Mini-Mental State Examination score by race and ethnic group. Beta coefficients and 95% confidence intervals (CIs) for associations between plasma biomarkers and 10-year atherosclerotic cardiovascular disease (ASCVD) risk on Mini-Mental State Examination (MMSE) scores, stratified by race and ethnic group. Models examined five biomarkers: Aβ42/40 (Model 1), p-tau217 (Model 2), p-tau181 (Model 3), neurofilament light chain (NfL) (Model 4), and apolipoprotein ε4 carrier status (*APOE* ε4) (Model 5). Each model included the 10-year ASCVD score too. Points represent point estimates, horizontal lines represent 95% CIs, and closed circles indicate statistically significant associations where the 95% CI does not cross zero (*p* < 0.05). Row A represents Non-Hispanic White participants, Row B represents Non-Hispanic Black participants, and Row C represents Hispanic participants.

### Race and ethnic stratification of dementia severity associations

Associations between plasma biomarkers and dementia severity measured by the CDR-SB varied across race and ethnic groups (Figure 3, Supplementary Tables 7–9). Among NHW participants (*N* = 858–883), ten-year ASCVD risk associations with CDR-SB were weak and did not reach statistical significance in most models, with β coefficients ranging from 0.11 (95% CI: −0.08 to 0.30; *p* = 0.251) to 0.24 (95% CI: 0.04 to 0.43; *p* = 0.017) (Figure 3A, Supplementary Table 7). However, *APOE* ε4 carrier status showed a significant association (*β* = 0.24; 95% CI: 0.04 to 0.43; *p* = 0.017). Among biomarkers, p-tau181 and Aβ42/40 demonstrated significant associations in NHW participants. p-tau181 was associated with worse dementia severity (β = 0.05; 95% CI: 0.02 to 0.07; *p* < 0.001), while Aβ42/40 showed an inverse association (*β* = −0.13; 95% CI: −0.22 to −0.05; *p* = 0.002). Plasma p-tau217 was also associated with increased severity (*β* = 0.05; 95% CI: 0.01 to 0.08; *p* = 0.019). NfL did not reach significance.

**Figure 3:**
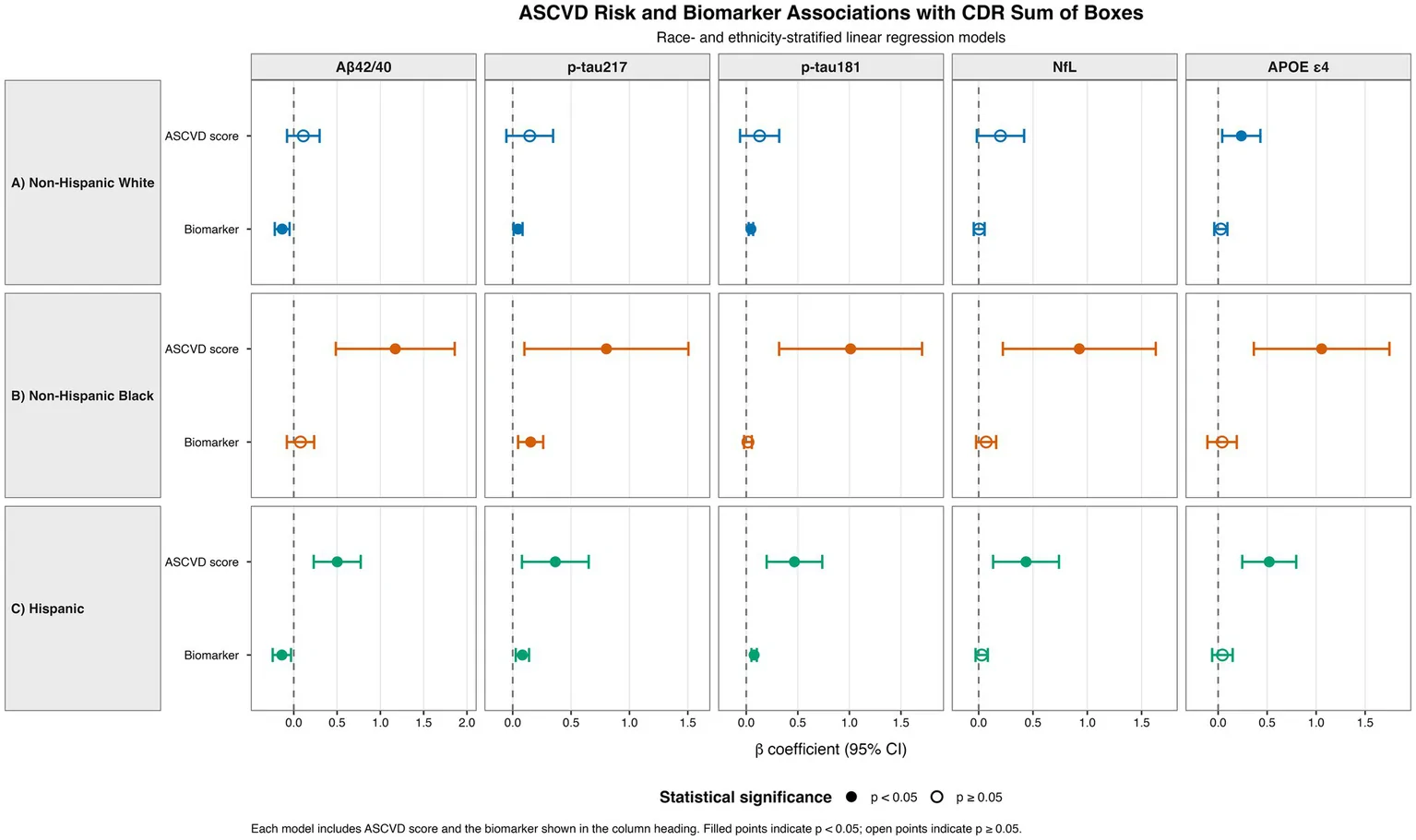
Forest plots of plasma biomarker and 10-year atherosclerotic cardiovascular disease (ASCVD) risk associations with Clinical Dementia Rating Sum of Boxes scores by race and ethnic group. Beta coefficients and 95% confidence intervals (CIs) for associations between plasma biomarkers and 10-year atherosclerotic cardiovascular disease (ASCVD) risk on Clinical Dementia Rating Sum of Boxes (CDR Sum of Boxes) scores, stratified by race and ethnic group. Models examined five biomarkers: Aβ42/40 (Model 1), p-tau217 (Model 2), p-tau181 (Model 3), neurofilament light chain (NfL) (Model 4), and apolipoprotein ε4 carrier status (*APOE* ε4) (Model 5). Each model included the 10-year ASCVD score too. Points represent point estimates, horizontal lines represent 95% CIs, and closed circles indicate statistically significant associations where the 95% CI does not cross zero (*p* < 0.05). Row A represents Non-Hispanic White participants, Row B represents Non-Hispanic Black participants, and Row C represents Hispanic participants.

NHB participants demonstrated higher estimate ten-year ASCVD regression coefficients with dementia severity relative to NHW participants (Figure 3B, Supplementary Table 8). Ten-year ASCVD risk *β* coefficients ranged from 0.80 (95% CI: 0.10 to 1.51; *p* = 0.025) in the p-tau217 model to 1.17 (95% CI: 0.49 to 1.86; *p* = 0.001) in the Aβ42/40 model. Among biomarkers, p-tau217 was significantly associated with CDR-SB (*β* = 0.15; 95% CI: 0.05 to 0.26; *p* = 0.005). Aβ42/40, p-tau181, NfL, and *APOE* ε4 carrier did not reach statistical significance in NHB participants.

Hispanic participants demonstrated ten-year ASCVD risk associations with dementia severity intermediate between NHW and NHB participants, with *β* coefficients ranging from 0.36 (95% CI: 0.08 to 0.65; *p* = 0.012) in the p-tau217 model to 0.52 (95% CI: 0.25 to 0.80; *p* < 0.001) in the *APOE* ε4 carrier model (Figure 3C, Supplementary Table 9). Increases in plasma p-tau181 demonstrated a significant association in Hispanic participants (*β* = 0.08; 95% CI: 0.05 to 0.10; *p* < 0.001). Aβ42/40 and p-tau217 also showed significant associations (*β* = −0.14; 95% CI: −0.24 to −0.03; *p* = 0.011 and *β* = 0.08; 95% CI: 0.03 to 0.14; *p* = 0.005, respectively). NfL and *APOE ε4* carrier status did not reach significance.

Across racial and ethnic groups, significant associations were observed for p-tau181 in NHW and Hispanic participants and for p-tau217 in NHB participants, whereas the magnitude of ten-year ASCVD risk associations varied across groups (Figures 3A–C).

## Discussion

In this large, community-based cohort enriched for racial and ethnic diversity, we observed that ten-year ASCVD risk and plasma AD biomarkers were independently associated with MCI. In addition, ten-year ASCVD risk modified the association between plasma p-tau181 and cognitive impairment, such that the relationship between p-tau181 and MCI had a greater magnitude of associations with increasing ten-year ASCVD risk. This interaction was not observed for p-tau217, Aβ42/40, NfL, or *APOE* ε4 status. Together, these findings suggest that cardiovascular risk burden may modify the clinical expression of selected AD-related biomarker signals, particularly p-tau181, prior to over dementia.

The observed interaction between ten-year ASCVD risk and p-tau181 suggests that ten-year ASCVD risk may influence the clinical expression of AD-related pathology captured by p-tau181 but not p-tau217. Several biological mechanisms may contribute to this relationship. CVD risk factors are associated with endothelial dysfunction, arterial stiffness, chronic systemic inflammation, and disruption of the blood–brain barrier, processes that increase neuronal vulnerability and impair the clearance of pathological proteins (Gorelick et al., 2011; Iadecola, 2013; Nation et al., 2019; Edwards et al., 2025). Vascular injury has also been linked to altered perivascular clearance pathways and reduced glymphatic function, which may promote the accumulation and propagation of pathological tau species (Iliff et al., 2012; Tarasoff-Conway et al., 2015; Rasmussen et al., 2018; Kang et al., 2026). These vascular mechanisms provide a biologically plausible framework through which cardiovascular risk may differentially influence circulating tau biomarkers.

Within this context, p-tau181 and p-tau217 both reflect AD-related biological processes and convey substantial overlapping information regarding amyloid pathology. However, p-tau217 has generally demonstrated stronger discrimination of AD pathology and closer associations with tau positron emission tomography than p-tau181, while both biomarkers have shown comparable ability to predict longitudinal Aβ accumulation in asymptomatic individuals (Palmqvist et al., 2020; Thijssen et al., 2021; Ashton et al., 2024; De Meyer et al., 2024). In contrast, p-tau181 elevations have been observed in association with more general neuronal injury and may be influenced by vascular-related processes, including blood–brain barrier dysfunction and impaired clearance (Wang et al., 2021; Rather et al., 2024). These vascular mechanisms may preferentially enhance tau phosphorylation or release detectable by p-tau181, providing a plausible explanation for why p-tau181, but not p-tau217, demonstrated an interaction with ten-year ASCVD risk in the present study. The differential interaction observed across tau isoforms highlights the importance of evaluating plasma tau biomarkers individually rather than assuming uniform biological behavior.

Despite statistically significant associations, model discrimination was modest. The AUC values from the logistic regression models were approximately 0.55. Accordingly, while ten-year ASCVD risk and plasma biomarkers capture meaningful associations with MCI at the population level, their utility for individual-level classification remains limited. The modest discrimination likely reflects substantial overlap in biomarker concentrations and ten-year ASCVD risk distributions across cognitive groups, a pattern that has been observed in other cross-sectional biomarker studies of early cognitive impairment (Albrecht et al., 2020; Kancheva et al., 2025).

These findings highlight an important distinction between biological association and clinical prediction. Although ten-year ASCVD risk and plasma AD biomarkers were associated with MCI, their ability to classify individuals was limited. As a result, these markers are likely most informative when interpreted alongside longitudinal biomarker data, neuroimaging measures, and detailed cognitive assessments rather than as stand-alone diagnostic tools (Villemagne et al., 2013; Hampel et al., 2023).

Prior studies have reported heterogeneous results, with some demonstrating additive associations between vascular burden and p-tau markers and others reporting attenuation after covariate adjustment (Rabin et al., 2019; Weigand et al., 2022). Differences in cohort composition, comorbidity burden, disease stage, and operationalization of cardiovascular risk may contribute to variability across studies. The use of a standardized ten-year ASCVD risk score provides a clinically interpretable summary of systemic vascular burden and future CVD risk, which also corroborates with increased risk for dementia (Arnett et al., 2019; Wong et al., 2022). Our findings extend this literature by demonstrating an interaction specifically for p-tau181. This observation highlights potential biological differences across plasma tau isoforms and raises the possibility that vascular risk may preferentially influence tau signals reflecting mixed vascular–neurodegenerative processes rather than amyloid-specific pathology.

Several limitations should be considered. First, the cross-sectional design precludes inference regarding temporality or causality between ten-year ASCVD risk, plasma biomarkers, and cognitive impairment. Second, ten-year ASCVD risk reflects an estimated ten-year cardiovascular risk based on pooled cohort equations rather than direct measures of subclinical atherosclerosis, which may introduce measurement imprecision (Yadlowsky et al., 2018). Third, demographic and clinical characteristics differed across the ten-year ASCVD risk distribution, with greater-risk groups comprising older individuals and a larger proportion of males. However, these ten-year ASCVD risk categories were presented for descriptive purposes only, whereas all primary analyses modeled ten-year ASCVD risk as a continuous predictor. The older age observed in increased ten-year ASCVD risk categories was expected because age is a major component of the pooled cohort equation used to estimate ten-year ASCVD risk. Future longitudinal studies will be better positioned to evaluate potential sex-specific survival or selection effects. Fourth, dementia cases were excluded, limiting inference to preclinical and early symptomatic stages of disease. Although cognitive diagnoses in HABS-HD were assigned using a diagnostic algorithm and consensus review, the MCI group may include individuals with heterogeneous or milder impairment, particularly among participants near the lower diagnostic threshold. Specifically, individuals with CDR-SB scores near 0.5 may represent a broader clinical range than those with more advanced impairment, and self- or informant-reported cognitive change may introduce variability. Therefore, while the cognitively unimpaired group is likely to represent a robustly unimpaired reference group, some misclassification within the MCI group is possible and may attenuate observed associations involving diagnostic group status.

Future studies with more complete longitudinal follow-up in HABS-HD will be important to determine whether ten-year ASCVD risk improves prediction of cognitive decline over time and whether cardiovascular risk modifies biomarker-associated cognitive trajectories. Such analyses will be particularly informative once longitudinal follow-up is sufficient across racial and ethnic groups. Future work incorporating amyloid positron emission tomography and other neuroimaging markers will also be important to determine whether amyloid status modifies the association between cardiovascular risk, plasma tau biomarkers, and cognitive outcomes. Because amyloid positron emission tomography is currently available in only a subset of HABS-HD participants, amyloid-stratified analyses were not included in the present baseline study to avoid substantial sample restriction and potential selection bias. As longitudinal follow-up becomes more complete and balanced across racial and ethnic groups, linear mixed-effects models will provide an important framework for determining whether ten-year ASCVD risk and plasma biomarkers jointly predict cognitive decline over time. In addition, although the ten-year ASCVD pooled cohort equation was intentionally evaluated as a composite measure of cumulative cardiovascular risk, future studies should investigate the relative contribution of individual cardiovascular risk factors and whether targeted modification of specific risk factors may preferentially reduce AD-related biomarker abnormalities and cognitive decline (Hayes et al., 2025a, 2026). Such work could ultimately inform more precise CVD prevention strategies for dementia risk reduction.

In conclusion, ten-year ASCVD risk and plasma AD biomarkers were independently associated with MCI in a racially and ethnically diverse cohort, and ten-year ASCVD risk modified the association between p-tau181 and cognitive impairment. These findings suggest that cardiovascular risk may influence the clinical relevance of specific AD biomarkers during the early symptomatic stages of disease. Longitudinal studies integrating vascular metrics with multimodal biomarkers will be important for clarifying the temporal and mechanistic relationships underlying these associations.

## Data Availability

Publicly available datasets were analyzed in this study. This data can be found at: https://loni.usc.edu/.
