# Supplementary material for: Plasma p-tau181 moderates the association between ten-year atherosclerotic cardiovascular disease risk and mild cognitive impairment – HABS-HD
Source: Front Aging Neurosci. 2026 Sep 28;18:1870150. doi: 10.3389/fnagi.2026.1870150 (PMC13655045; doi:10.3389/fnagi.2026.1870150)
Supplement: Supplementary file 1 [file Table_1.docx]

**Supplemental Table 1. Logistic Regression Models Examining Associations Between Plasma Biomarkers, Ten-year Atherosclerotic Cardiovascular Disease Risk, and Mild Cognitive Impairment Adjusted for Estimated Glomerular Filtration Rate (Sensitivity Analysis).** Sensitivity analysis of logistic regression models examining the independent associations of five plasma biomarkers and ten-year atherosclerotic cardiovascular disease (ASCVD) risk with mild cognitive impairment (MCI) status, with the addition of estimated glomerular filtration rate (eGFR) as a covariate to account for renal function. Each model includes one plasma biomarker, ten-year ASCVD risk score, and eGFR. Model 1 includes Aβ42/40 (log2-transformed); Model 2 includes p-tau217 (log2-transformed); Model 3 includes p-tau181 (log2-transformed); Model 4 includes neurofilament light chain (NfL, log2-transformed); and Model 5 includes apolipoprotein E ε4 carrier status (APOE ε4, binary). Odds ratios (OR) represent the change in odds of MCI per unit increase in the biomarker or per unit increase in eGFR, or the presence of APOE ε4 carrier status. Sample sizes (N) are provided for each model and vary due to missing biomarker and eGFR data. R² Tjur represents the coefficient of determination for logistic regression. AIC (Akaike Information Criterion) and AUC (area under the receiver operating characteristic curve) assess model fit. VIF (variance inflation factor) tests for multicollinearity. P-values <0.05 indicate statistical significance.

| **Variable** | **P value** | **OR (95% CI)** | **R^2^ Tjur** | **AIC** | **AUC** | **VIF** |
| --- | --- | --- | --- | --- | --- | --- |
| **Model 1: Aβ42/40 + Ten-year ASCVD Risk Score + eGFR (N=1990)** |  |  | 0.009 | 2002.069 | 0.563 |  |
| Aβ42/40 (log2) | 0.094 | 0.78 (0.58 to 1.04) |  |  |  | 1.004 |
| eGFR | 0.014 | 1.01 (1.00 to 1.02) |  |  |  | 1.180 |
| Ten-year ASCVD Risk Score | 0.001 | 3.89 (1.78 to 8.39) |  |  |  | 1.181 |
| **Model 2: p-Tau217 + Ten-year ASCVD Risk Score + eGFR (N=2038)** |  |  | 0.013 | 2046.986 | 0.580 |  |
| p-Tau217 (log2) | 0.001 | 1.30 (1.12 to 1.50) |  |  |  | 1.230 |
| eGFR | 0.001 | 1.01 (1.01 to 1.02) |  |  |  | 1.317 |
| Ten-year ASCVD Risk Score | 0.002 | 3.36 (1.54 to 7.27) |  |  |  | 1.212 |
| **Model 3: p-Tau181 + Ten-year ASCVD Risk Score + eGFR (N=1971)** |  |  | 0.030 | 1955.317 | 0.613 |  |
| p-Tau181 (log2) | <0.001 | 1.26 (1.18 to 1.35) |  |  |  | 1.001 |
| eGFR | 0.009 | 1.01 (1.00 to 1.02) |  |  |  | 1.173 |
| Ten-year ASCVD Risk Score | 0.001 | 3.60 (1.64 to 7.81) |  |  |  | 1.173 |
| **Model 4: NfL + Ten-year ASCVD Risk Score + eGFR (N=2038)** |  |  | 0.008 | 2057.202 | 0.564 |  |
| NfL (log2) | 0.269 | 0.91 (0.77 to 1.07) |  |  |  | 1.406 |
| eGFR | 0.049 | 1.01 (1.00 to 1.02) |  |  |  | 1.347 |
| Ten-year ASCVD Risk Score | <0.001 | 4.87 (2.18 to 10.79) |  |  |  | 1.302 |
| **Model 5: APOE ε4 + Ten-year ASCVD Risk Score + eGFR (N=2028)** |  |  | 0.010 | 2048.992 | 0.569 |  |
| APOE ε4 | 0.032 | 1.30 (1.02 to 1.65) |  |  |  | 1.012 |
| eGFR | 0.010 | 1.01 (1.00 to 1.02) |  |  |  | 1.177 |
| Ten-year ASCVD Risk Score | <0.001 | 4.75 (2.19 to 10.20) |  |  |  | 1.189 |

**Supplemental Table 2. Linear Regression Interaction Models Examining ASCVD Risk × Plasma Biomarker Interactions in Relation to Global Cognition.** Linear regression models with interaction terms examining whether the associations between plasma biomarkers and global cognitive function are modified by ten-year atherosclerotic cardiovascular disease (ASCVD) risk, as assessed by Mini-Mental State Examination (MMSE) total score. Each model includes one plasma biomarker, ten-year ASCVD risk score, and the interaction term (ASCVD × biomarker). Model 1 includes Aβ42/40 (log2-transformed); Model 2 includes p-tau217 (log2-transformed); Model 3 includes p-tau181 (log2-transformed); Model 4 includes neurofilament light chain (NfL, log2-transformed); and Model 5 includes apolipoprotein E ε4 carrier status (APOE ε4, binary). β coefficients represent the change in MMSE score per unit increase in the respective predictor. Sample sizes (N) are provided for each model and vary due to missing biomarker data. R², coefficient of determination; Adjusted R², R² adjusted for number of predictors; AIC, Akaike Information Criterion; VIF, variance inflation factor (tests for multicollinearity).

| **Variable** | **P value** | **β (95% CI)** | **R² / Adjusted R²** | **AIC** | **VIF** |
| --- | --- | --- | --- | --- | --- |
| **Model 1: Aβ42/40 + Ten-year ASCVD Risk Score + Interaction (N=2231)** |  |  | 0.014 / 0.012 | 10436.101 |  |
| Aβ42/40 (log2) | 0.314 | 0.21 (-0.20 to 0.62) |  |  | 2.331 |
| Ten-year ASCVD Risk Score | 0.701 | -1.61 (-9.84 to 6.62) |  |  | 131.866 |
| Ten-year ASCVD Risk Score x Aβ42/40 (log2) | 0.945 | 0.06 (-1.77 to 1.90) |  |  | 134.508 |
| **Model 2: p-Tau217 + Ten-year ASCVD Risk Score + Interaction (N=2282)** |  |  | 0.024 / 0.023 | 10643.102 |  |
| p-Tau217 (log2) | <0.001 | 0.47 (0.26 to 0.67) |  |  | 2.451 |
| Ten-year ASCVD Risk Score | <0.001 | -3.71 (-5.09 to -2.33) |  |  | 3.816 |
| Ten-year ASCVD Risk Score x p-Tau217 (log2) | 0.052 | -0.82 (-1.65 to 0.01) |  |  | 3.908 |
| **Model 3: p-Tau181 + Ten-year ASCVD Risk Score + Interaction (N=2213)** |  |  | 0.015 / 0.014 | 10288.131 |  |
| p-Tau181 (log2) | 0.090 | -0.08 (-0.18 to 0.01) |  |  | 2.302 |
| Ten-year ASCVD Risk Score | <0.001 | -2.11 (-3.20 to -1.03) |  |  | 2.350 |
| Ten-year ASCVD Risk Score x p-Tau181 (log2) | 0.620 | 0.12 (-0.34 to 0.57) |  |  | 3.796 |
| **Model 4: NfL + Ten-year ASCVD Risk Score + Interaction (N=2282)** |  |  | 0.014 / 0.013 | 10666.319 |  |
| NfL (log2) | 0.303 | 0.11 (-0.10 to 0.31) |  |  | 2.402 |
| Ten-year ASCVD Risk Score | 0.832 | -0.40 (-4.08 to 3.28) |  |  | 26.975 |
| Ten-year ASCVD Risk Score x NfL (log2) | 0.346 | -0.42 (-1.29 to 0.45) |  |  | 32.570 |
| **Model 5: APOE ε4 + Ten-year ASCVD Risk Score + Interaction (N=2250)** |  |  | 0.021 / 0.020 | 10499.616 |  |
| APOE ε4 | 0.065 | 0.33 (-0.02 to 0.69) |  |  | 2.336 |
| Ten-year ASCVD Risk Score | <0.001 | -2.16 (-2.97 to -1.36) |  |  | 1.276 |
| Ten-year ASCVD Risk Score x APOE ε4 | 0.370 | 0.80 (-0.95 to 2.55) |  |  | 2.510 |

**Supplemental Table 3. Linear Regression Interaction Models Examining ASCVD Risk × Plasma Biomarker Interactions in Relation to Dementia Severity.** Linear regression models with interaction terms examining whether the associations between plasma biomarkers and dementia severity are modified by ten-year atherosclerotic cardiovascular disease (ASCVD) risk, as assessed by Clinical Dementia Rating (CDR) Sum of Boxes. Each model includes one plasma biomarker, ten-year ASCVD risk score, and the interaction term (ASCVD × biomarker). Model 1 includes Aβ42/40 (log2-transformed); Model 2 includes p-tau217 (log2-transformed); Model 3 includes p-tau181 (log2-transformed); Model 4 includes neurofilament light chain (NfL, log2-transformed); and Model 5 includes apolipoprotein E ε4 carrier status (APOE ε4, binary). β coefficients represent the change in CDR Sum of Boxes per unit increase in the respective predictor; positive values indicate worse dementia severity. Sample sizes (N) are provided for each model and vary due to missing biomarker data. R², coefficient of determination; Adjusted R², R² adjusted for number of predictors; AIC, Akaike Information Criterion; VIF, variance inflation factor (tests for multicollinearity).

| **Variable** | **P value** | **β (95% CI)** | **R² / Adjusted R²** | **AIC** | **VIF** |
| --- | --- | --- | --- | --- | --- |
| **Model 1: Aβ42/40 + Ten-year ASCVD Risk Score + Interaction (N=2240)** |  |  | 0.012 / 0.010 | 4160.476 |  |
| Aβ42/40 (log2) | 0.545 | -0.03 (-0.13 to 0.07) |  |  | 2.329 |
| Ten-year ASCVD Risk Score | 0.157 | -1.45 (-3.45 to 0.56) |  |  | 132.021 |
| Ten-year ASCVD Risk Score x Aβ42/40 (log2) | 0.082 | -0.40 (-0.84 to 0.05) |  |  | 134.683 |
| **Model 2: p-Tau217 + Ten-year ASCVD Risk Score + Interaction (N=2292)** |  |  | 0.011 / 0.010 | 4319.960 |  |
| p-Tau217 (log2) | 0.067 | 0.05 (-0.00 to 0.10) |  |  | 2.451 |
| Ten-year ASCVD Risk Score | 0.082 | 0.30 (-0.04 to 0.65) |  |  | 3.818 |
| Ten-year ASCVD Risk Score x p-Tau217 (log2) | 0.862 | 0.02 (-0.19 to 0.22) |  |  | 3.904 |
| **Model 3: p-Tau181 + Ten-year ASCVD Risk Score + Interaction (N=2223)** |  |  | 0.042 / 0.040 | 4101.482 |  |
| p-Tau181 (log2) | 0.137 | 0.02 (-0.01 to 0.04) |  |  | 2.299 |
| Ten-year ASCVD Risk Score | 0.154 | -0.19 (-0.46 to 0.07) |  |  | 2.355 |
| Ten-year ASCVD Risk Score x p-Tau181 (log2) | <0.001 | 0.27 (0.16 to 0.39) |  |  | 3.797 |
| **Model 4: NfL + Ten-year ASCVD Risk Score + Interaction (N=2292)** |  |  | 0.008 / 0.006 | 4327.409 |  |
| NfL (log2) | 0.289 | -0.03 (-0.08 to 0.02) |  |  | 2.396 |
| Ten-year ASCVD Risk Score | 0.953 | 0.03 (-0.88 to 0.94) |  |  | 26.976 |
| Ten-year ASCVD Risk Score x NfL (log2) | 0.424 | 0.09 (-0.13 to 0.30) |  |  | 32.557 |
| **Model 5: APOE ε4 + Ten-year ASCVD Risk Score + Interaction (N=2260)** |  |  | 0.009 / 0.007 | 4277.439 |  |
| APOE ε4 | 0.200 | 0.06 (-0.03 to 0.15) |  |  | 2.336 |
| Ten-year ASCVD Risk Score | <0.001 | 0.40 (0.20 to 0.60) |  |  | 1.274 |
| Ten-year ASCVD Risk Score x APOE ε4 | 0.665 | -0.10 (-0.53 to 0.34) |  |  | 2.510 |

**Supplemental Table 4. Associations Between Plasma Biomarkers, Ten-year Atherosclerotic Cardiovascular Disease Risk, and Mini-Mental State Examination Score in Non-Hispanic White Participants.** Linear regression models examining the independent associations of five plasma biomarkers and ten-year atherosclerotic cardiovascular disease (ASCVD) risk with Mini-Mental State Examination (MMSE) total score in the Non-Hispanic White participants. Each model includes one plasma biomarker alongside ten-year ASCVD risk score as a covariate. Model 1 includes Aβ42/40 (log2-transformed); Model 2 includes p-tau217 (log2-transformed); Model 3 includes p-tau181 (log2-transformed); Model 4 includes neurofilament light chain (NfL, log2-transformed); and Model 5 includes apolipoprotein E ε4 carrier status (APOE ε4, binary). β coefficients represent the change in MMSE score per unit increase in the biomarker (or presence of APOE ε4 carrier status). P-values <0.05 indicate statistical significance. 95% CI, 95% confidence interval; R², coefficient of determination; Adjusted R², R² adjusted for number of predictors; AIC, Akaike Information Criterion. Sample sizes (N) are provided for each model and vary due to missing biomarker data.

| **Variable** | **P value** | **β (95% CI)** | **R² / Adjusted R²** | **AIC** |
| --- | --- | --- | --- | --- |
| **Model 1: Aβ42/40 + Ten-year ASCVD Risk Score (N=854)** |  |  | 0.060 / 0.057 | 2555.906 |
| Aβ42/40 (log2) | 0.066 | 0.19 (-0.01 to 0.40) |  |  |
| Ten-year ASCVD Risk Score | <0.001 | -1.62 (-2.08 to -1.17) |  |  |
| **Model 2: p-Tau217 + Ten-year ASCVD Risk Score (N=877)** |  |  | 0.060 / 0.058 | 2678.750 |
| p-Tau217 (log2) | 0.621 | -0.02 (-0.12 to 0.07) |  |  |
| Ten-year ASCVD Risk Score | <0.001 | -1.73 (-2.21 to -1.25) |  |  |
| **Model 3: p-Tau181 + Ten-year ASCVD Risk Score (N=852)** |  |  | 0.061 / 0.059 | 2580.374 |
| p-Tau181 (log2) | 0.088 | -0.05 (-0.10 to 0.01) |  |  |
| Ten-year ASCVD Risk Score | <0.001 | -1.68 (-2.15 to -1.22) |  |  |
| **Model 4: NfL + Ten-year ASCVD Risk Score (N=877)** |  |  | 0.062 / 0.060 | 2676.477 |
| NfL (log2) | 0.113 | 0.10 (-0.02 to 0.22) |  |  |
| Ten-year ASCVD Risk Score | <0.001 | -1.96 (-2.48 to -1.43) |  |  |
| **Model 5: APOE ε4 + Ten-year ASCVD Risk Score (N=866)** |  |  | 0.060 / 0.058 | 2649.806 |
| APOE ε4 | 0.804 | 0.02 (-0.14 to 0.18) |  |  |
| Ten-year ASCVD Risk Score | <0.001 | -1.77 (-2.24 to -1.30) |  |  |

**Supplemental Table 5. Associations Between Plasma Biomarkers, Ten-year Atherosclerotic Cardiovascular Disease Risk, and Mini-Mental State Examination Score in Non-Hispanic White Participants.** Linear regression models examining the independent associations of five plasma biomarkers and ten-year atherosclerotic cardiovascular disease (ASCVD) risk with Mini-Mental State Examination (MMSE) total score in the Non-Hispanic White participants. Each model includes one plasma biomarker alongside ten-year ASCVD risk score as a covariate. Model 1 includes Aβ42/40 (log2-transformed); Model 2 includes p-tau217 (log2-transformed); Model 3 includes p-tau181 (log2-transformed); Model 4 includes neurofilament light chain (NfL, log2-transformed); and Model 5 includes apolipoprotein E ε4 carrier status (APOE ε4, binary). β coefficients represent the change in MMSE score per unit increase in the biomarker (or presence of APOE ε4 carrier status). P-values <0.05 indicate statistical significance. 95% CI, 95% confidence interval; R², coefficient of determination; Adjusted R², R² adjusted for number of predictors; AIC, Akaike Information Criterion. Sample sizes (N) are provided for each model and vary due to missing biomarker data.

| **Variable** | **P value** | **β (95% CI)** | **R² / Adjusted R²** | **AIC** |
| --- | --- | --- | --- | --- |
| **Model 1: Aβ42/40 + Ten-year ASCVD Risk Score (N=451)** |  |  | 0.024 / 0.020 | 1829.647 |
| Aβ42/40 (log2) | 0.127 | 0.29 (-0.08 to 0.65) |  |  |
| Ten-year ASCVD Risk Score | 0.004 | -2.35 (-3.95 to -0.75) |  |  |
| **Model 2: p-Tau217 + Ten-year ASCVD Risk Score (N=472)** |  |  | 0.023 / 0.019 | 1903.087 |
| p-Tau217 (log2) | 0.293 | -0.13 (-0.38 to 0.11) |  |  |
| Ten-year ASCVD Risk Score | 0.005 | -2.27 (-3.87 to -0.67) |  |  |
| **Model 3: p-Tau181 + Ten-year ASCVD Risk Score (N=471)** |  |  | 0.043 / 0.039 | 1879.389 |
| p-Tau181 (log2) | 0.001 | -0.14 (-0.22 to -0.05) |  |  |
| Ten-year ASCVD Risk Score | 0.006 | -2.14 (-3.68 to -0.60) |  |  |
| **Model 4: NfL + Ten-year ASCVD Risk Score (N=472)** |  |  | 0.041 / 0.037 | 1894.123 |
| NfL (log2) | 0.002 | -0.33 (-0.54 to -0.13) |  |  |
| Ten-year ASCVD Risk Score | 0.018 | -1.90 (-3.48 to -0.33) |  |  |
| **Model 5: APOE ε4 + Ten-year ASCVD Risk Score (N=469)** |  |  | 0.021 / 0.017 | 1893.030 |
| APOE ε4 | 0.762 | 0.05 (-0.29 to 0.39) |  |  |
| Ten-year ASCVD Risk Score | 0.002 | -2.47 (-4.03 to -0.91) |  |  |

**Supplemental Table 6. Associations Between Plasma Biomarkers, Ten-year Atherosclerotic Cardiovascular Disease Risk, and Mini-Mental State Examination Score in Hispanic Participants.** Linear regression models examining the independent associations of five plasma biomarkers and ten-year atherosclerotic cardiovascular disease (ASCVD) risk with Mini-Mental State Examination (MMSE) total score in the Hispanic participants. Each model includes one plasma biomarker alongside ten-year ASCVD risk score as a covariate. Model 1 includes Aβ42/40 (log2-transformed); Model 2 includes p-tau217 (log2-transformed); Model 3 includes p-tau181 (log2-transformed); Model 4 includes neurofilament light chain (NfL, log2-transformed); and Model 5 includes apolipoprotein E ε4 carrier status (APOE ε4, binary). β coefficients represent the change in MMSE score per unit increase in the biomarker (or presence of APOE ε4 carrier status). P-values <0.05 indicate statistical significance. 95% CI, 95% confidence interval; R², coefficient of determination; Adjusted R², R² adjusted for number of predictors; AIC, Akaike Information Criterion. Sample sizes (N) are provided for each model and vary due to missing biomarker data.

| **Variable** | **P value** | **β (95% CI)** | **R² / Adjusted R²** | **AIC** |
| --- | --- | --- | --- | --- |
| **Model 1: Aβ42/40 + Ten-year ASCVD Risk Score (N=926)** |  |  | 0.033 / 0.031 | 4656.697 |
| Aβ42/40 (log2) | 0.205 | 0.33 (-0.18 to 0.84) |  |  |
| Ten-year ASCVD Risk Score | <0.001 | -3.61 (-4.91 to -2.30) |  |  |
| **Model 2: p-Tau217 + Ten-year ASCVD Risk Score (N=933)** |  |  | 0.034 / 0.032 | 4698.648 |
| p-Tau217 (log2) | 0.735 | 0.05 (-0.23 to 0.32) |  |  |
| Ten-year ASCVD Risk Score | <0.001 | -3.90 (-5.29 to -2.52) |  |  |
| **Model 3: p-Tau181 + Ten-year ASCVD Risk Score (N=890)** |  |  | 0.036 / 0.034 | 4460.201 |
| p-Tau181 (log2) | 0.057 | -0.12 (-0.25 to 0.00) |  |  |
| Ten-year ASCVD Risk Score | <0.001 | -3.57 (-4.88 to -2.26) |  |  |
| **Model 4: NfL + Ten-year ASCVD Risk Score (N=933)** |  |  | 0.037 / 0.035 | 4696.028 |
| NfL (log2) | 0.099 | -0.23 (-0.50 to 0.04) |  |  |
| Ten-year ASCVD Risk Score | <0.001 | -3.26 (-4.72 to -1.80) |  |  |
| **Model 5: APOE ε4 + Ten-year ASCVD Risk Score (N=915)** |  |  | 0.037 / 0.035 | 4607.933 |
| APOE ε4 | 0.308 | 0.26 (-0.24 to 0.76) |  |  |
| Ten-year ASCVD Risk Score | <0.001 | -3.83 (-5.15 to -2.51) |  |  |

**Supplemental Table 7. Associations Between Plasma Biomarkers, Ten-year Atherosclerotic Cardiovascular Disease Risk, and Clinical Dementia Rating Sum of Boxes Score in Non-Hispanic White Participants.** Linear regression models examining the independent associations of five plasma biomarkers and ten-year atherosclerotic cardiovascular disease (ASCVD) risk with Mini-Clinical Dementia Rating (CDR) Sum of Boxes total score in the Non-Hispanic White participants. Each model includes one plasma biomarker alongside ten-year ASCVD risk score as a covariate. Model 1 includes Aβ42/40 (log2-transformed); Model 2 includes p-tau217 (log2-transformed); Model 3 includes p-tau181 (log2-transformed); Model 4 includes neurofilament light chain (NfL, log2-transformed); and Model 5 includes apolipoprotein E ε4 carrier status (APOE ε4, binary). β coefficients represent the change in CDR Sum of Boxes score per unit increase in the biomarker (or presence of APOE ε4 carrier status). P-values <0.05 indicate statistical significance. 95% CI, 95% confidence interval; R², coefficient of determination; Adjusted R², R² adjusted for number of predictors; AIC, Akaike Information Criterion. Sample sizes (N) are provided for each model and vary due to missing biomarker data.

| **Variable** | **P value** | **β (95% CI)** | **R² / Adjusted R²** | **AIC** |
| --- | --- | --- | --- | --- |
| **Model 1: Aβ42/40 + Ten-year ASCVD Risk Score (N=859)** |  |  | 0.013 / 0.011 | 1050.088 |
| Aβ42/40 (log2) | 0.002 | -0.13 (-0.22 to -0.05) |  |  |
| Ten-year ASCVD Risk Score | 0.251 | 0.11 (-0.08 to 0.30) |  |  |
| **Model 2: p-Tau217 + Ten-year ASCVD Risk Score (N=883)** |  |  | 0.011 / 0.009 | 1148.943 |
| p-Tau217 (log2) | 0.019 | 0.05 (0.01 to 0.08) |  |  |
| Ten-year ASCVD Risk Score | 0.154 | 0.15 (-0.05 to 0.35) |  |  |
| **Model 3: p-Tau181 + Ten-year ASCVD Risk Score (N=858)** |  |  | 0.022 / 0.019 | 1069.575 |
| p-Tau181 (log2) | <0.001 | 0.05 (0.02 to 0.07) |  |  |
| Ten-year ASCVD Risk Score | 0.180 | 0.13 (-0.06 to 0.32) |  |  |
| **Model 4: NfL + Ten-year ASCVD Risk Score (N=883)** |  |  | 0.005 / 0.003 | 1154.389 |
| NfL (log2) | 0.853 | 0.00 (-0.05 to 0.05) |  |  |
| Ten-year ASCVD Risk Score | 0.071 | 0.20 (-0.02 to 0.42) |  |  |
| **Model 5: APOE ε4 + Ten-year ASCVD Risk Score (N=872)** |  |  | 0.007 / 0.005 | 1132.112 |
| APOE ε4 | 0.428 | 0.03 (-0.04 to 0.09) |  |  |
| Ten-year ASCVD Risk Score | 0.017 | 0.24 (0.04 to 0.43) |  |  |

**Supplemental Table 8. Associations Between Plasma Biomarkers, Ten-year Atherosclerotic Cardiovascular Disease Risk, and Clinical Dementia Rating Sum of Boxes Score in Non-Hispanic Black Participants.** Linear regression models examining the independent associations of five plasma biomarkers and ten-year atherosclerotic cardiovascular disease (ASCVD) risk with Mini-Clinical Dementia Rating (CDR) Sum of Boxes total score in the Non-Hispanic Black participants. Each model includes one plasma biomarker alongside ten-year ASCVD risk score as a covariate. Model 1 includes Aβ42/40 (log2-transformed); Model 2 includes p-tau217 (log2-transformed); Model 3 includes p-tau181 (log2-transformed); Model 4 includes neurofilament light chain (NfL, log2-transformed); and Model 5 includes apolipoprotein E ε4 carrier status (APOE ε4, binary). β coefficients represent the change in CDR Sum of Boxes score per unit increase in the biomarker (or presence of APOE ε4 carrier status). P-values <0.05 indicate statistical significance. 95% CI, 95% confidence interval; R², coefficient of determination; Adjusted R², R² adjusted for number of predictors; AIC, Akaike Information Criterion. Sample sizes (N) are provided for each model and vary due to missing biomarker data.

| **Variable** | **P value** | **β (95% CI)** | **R² / Adjusted R²** | **AIC** |
| --- | --- | --- | --- | --- |
| **Model 1: Aβ42/40 + Ten-year ASCVD Risk Score (N=452)** |  |  | 0.026 / 0.021 | 1068.225 |
| Aβ42/40 (log2) | 0.332 | 0.08 (-0.08 to 0.24) |  |  |
| Ten-year ASCVD Risk Score | 0.001 | 1.17 (0.49 to 1.86) |  |  |
| **Model 2: p-Tau217 + Ten-year ASCVD Risk Score (N=473)** |  |  | 0.035 / 0.031 | 1130.308 |
| p-Tau217 (log2) | 0.005 | 0.15 (0.05 to 0.26) |  |  |
| Ten-year ASCVD Risk Score | 0.025 | 0.80 (0.10 to 1.51) |  |  |
| **Model 3: p-Tau181 + Ten-year ASCVD Risk Score (N=472)** |  |  | 0.021 / 0.017 | 1131.979 |
| p-Tau181 (log2) | 0.393 | 0.02 (-0.02 to 0.05) |  |  |
| Ten-year ASCVD Risk Score | 0.004 | 1.01 (0.32 to 1.70) |  |  |
| **Model 4: NfL + Ten-year ASCVD Risk Score (N=473)** |  |  | 0.023 / 0.019 | 1136.020 |
| NfL (log2) | 0.143 | 0.07 (-0.02 to 0.16) |  |  |
| Ten-year ASCVD Risk Score | 0.010 | 0.93 (0.22 to 1.63) |  |  |
| **Model 5: APOE ε4 + Ten-year ASCVD Risk Score (N=470)** |  |  | 0.019 / 0.015 | 1132.919 |
| APOE ε4 | 0.598 | 0.04 (-0.11 to 0.19) |  |  |
| Ten-year ASCVD Risk Score | 0.003 | 1.06 (0.36 to 1.75) |  |  |

**Supplemental Table 9. Associations Between Plasma Biomarkers, Ten-year Atherosclerotic Cardiovascular Disease Risk, and Clinical Dementia Rating Sum of Boxes Score in Hispanic Participants.** Linear regression models examining the independent associations of five plasma biomarkers and ten-year atherosclerotic cardiovascular disease (ASCVD) risk with Mini-Clinical Dementia Rating (CDR) Sum of Boxes total score in the Hispanic participants. Each model includes one plasma biomarker alongside ten-year ASCVD risk score as a covariate. Model 1 includes Aβ42/40 (log2-transformed); Model 2 includes p-tau217 (log2-transformed); Model 3 includes p-tau181 (log2-transformed); Model 4 includes neurofilament light chain (NfL, log2-transformed); and Model 5 includes apolipoprotein E ε4 carrier status (APOE ε4, binary). β coefficients represent the change in CDR Sum of Boxes score per unit increase in the biomarker (or presence of APOE ε4 carrier status). P-values <0.05 indicate statistical significance. 95% CI, 95% confidence interval; R², coefficient of determination; Adjusted R², R² adjusted for number of predictors; AIC, Akaike Information Criterion. Sample sizes (N) are provided for each model and vary due to missing biomarker data.

| **Variable** | **P value** | **β (95% CI)** | **R² / Adjusted R²** | **AIC** |
| --- | --- | --- | --- | --- |
| **Model 1: Aβ42/40 + Ten-year ASCVD Risk Score (N=929)** |  |  | 0.022 / 0.019 | 1753.916 |
| Aβ42/40 (log2) | 0.011 | -0.14 (-0.24 to -0.03) |  |  |
| Ten-year ASCVD Risk Score | <0.001 | 0.50 (0.23 to 0.77) |  |  |
| **Model 2: p-Tau217 + Ten-year ASCVD Risk Score (N=936)** |  |  | 0.023 / 0.020 | 1761.617 |
| p-Tau217 (log2) | 0.005 | 0.08 (0.03 to 0.14) |  |  |
| Ten-year ASCVD Risk Score | 0.012 | 0.36 (0.08 to 0.65) |  |  |
| **Model 3: p-Tau181 + Ten-year ASCVD Risk Score (N=893)** |  |  | 0.050 / 0.047 | 1653.458 |
| p-Tau181 (log2) | <0.001 | 0.08 (0.05 to 0.10) |  |  |
| Ten-year ASCVD Risk Score | 0.001 | 0.47 (0.20 to 0.74) |  |  |
| **Model 4: NfL + Ten-year ASCVD Risk Score (N=936)** |  |  | 0.015 / 0.013 | 1768.740 |
| NfL (log2) | 0.336 | 0.03 (-0.03 to 0.08) |  |  |
| Ten-year ASCVD Risk Score | 0.005 | 0.44 (0.13 to 0.74) |  |  |
| **Model 5: APOE ε4 + Ten-year ASCVD Risk Score (N=918)** |  |  | 0.015 / 0.013 | 1743.364 |
| APOE ε4 | 0.415 | 0.04 (-0.06 to 0.15) |  |  |
| Ten-year ASCVD Risk Score | <0.001 | 0.52 (0.25 to 0.80) |  |  |
